# Mannose and Lactobionic Acid in Nasal Vaccination: Enhancing Antigen Delivery via C-Type Lectin Receptors

**DOI:** 10.3390/pharmaceutics16101308

**Published:** 2024-10-08

**Authors:** Mariana Colaço, Maria T. Cruz, Luís Pereira de Almeida, Olga Borges

**Affiliations:** 1 CNC-UC—Center for Neuroscience and Cell Biology, University of Coimbra, 3004-504 Coimbra, Portugal; mariana.colaco@cnc.uc.pt (M.C.); trosete@ff.uc.pt (M.T.C.); luispa@cnc.uc.pt (L.P.d.A.); 2 CIBB—Center for Innovative Biomedicine and Biotechnology, University of Coimbra, 3004-504 Coimbra, Portugal; 3 Faculty of Pharmacy, University of Coimbra, 3000-548 Coimbra, Portugal

**Keywords:** mucosal vaccines, nasal immunization, antigen-presenting cells, C-type lectin receptors, mannose receptor, macrophage galactose-type lectin receptor, vaccine development, lactobionic acid

## Abstract

Background/Objectives: Nasal vaccines are a promising strategy for enhancing mucosal immune responses and preventing diseases at mucosal sites by stimulating the secretion of secretory IgA, which is crucial for early pathogen neutralization. However, designing effective nasal vaccines is challenging due to the complex immunological mechanisms in the nasal mucosa, which must balance protection and tolerance against constant exposure to inhaled pathogens. The nasal route also presents unique formulation and delivery hurdles, such as the mucous layer hindering antigen penetration and immune cell access. Methods: This review focuses on cutting-edge approaches to enhance nasal vaccine delivery, particularly those targeting C-type lectin receptors (CLRs) like the mannose receptor and macrophage galactose-type lectin (MGL) receptor. It elucidates the roles of these receptors in antigen recognition and uptake by antigen-presenting cells (APCs), providing insights into optimizing vaccine delivery. Results: While a comprehensive examination of targeted glycoconjugate vaccine development is outside the scope of this study, we provide key examples of glycan-based ligands, such as lactobionic acid and mannose, which can selectively target CLRs in the nasal mucosa. Conclusions: With the rise of new viral infections, this review aims to facilitate the design of innovative vaccines and equip researchers, clinicians, and vaccine developers with the knowledge to enhance immune defenses against respiratory pathogens, ultimately protecting public health.

## 1. Introduction

Vaccination stands as a remarkable achievement in global health, saving millions of lives annually. By collaborating harmoniously with the body’s intrinsic immune defenses, vaccines diminish the likelihood of contracting diseases while strengthening the immune system [1]. Once a vaccine is administered, the body’s immunological system is stimulated, orchestrating a robust response. This immune response is marked by the rapid activation of the innate immune system, which promptly detects and responds to the introduced foreign elements, serving as the initial line of defense. Key innate immune cells, such as macrophages and dendritic cells (DCs), engulf, process, and present the antigens to the adaptive immune system, communicating a potential threat. In response, the adaptive immune system initiates a highly specific reaction: B cells produce antibodies that specifically target the pathogen’s antigens, while T cells play a central role in coordinating the cellular immune response, eliminating infected cells. The vaccine essentially acts as an “instructive session” for the immune system, allowing it to develop a memory of the pathogen’s characteristics without causing the actual illness. In the present era, a diverse array of vaccines exists, protecting against over 20 life-threatening illnesses, enabling individuals across all age groups to lead extended and more robust lives [2]. The act of immunization presently prevents 3.5 to 5 million deaths each year, counteracting diseases like tetanus, pertussis, influenza, diphtheria, and measles [3]. Immunization forms a pivotal cornerstone of primary healthcare, and it is unequivocally acknowledged as an intrinsic human right. Moreover, it stands among the wisest investments in healthcare resources. Additionally, as demonstrated by the COVID-19 pandemic, vaccines play an indispensable role in preventing and managing outbreaks of infectious diseases.

In recent years, the landscape of vaccine development has witnessed remarkable advancements, particularly in the field of parenteral administration. Injectable formulations, supported by innovative technologies, such as subunit vaccines and nucleic acid vaccines, have achieved substantial success in combating infectious diseases. Subunit vaccines, for instance, use purified components of pathogens to elicit strong immune responses while minimizing the risk of adverse effects associated with whole pathogens. On the other hand, nucleic acid vaccines, including mRNA and DNA vaccines, have gained considerable attention for their ability to instruct cells to produce antigenic proteins, triggering robust immune reactions [4]. The progress achieved in parenteral vaccine administration has been truly pioneering, leading to the development of vaccines against previously challenging targets. An exceptional demonstration of this achievement is the rapid development and implementation of COVID-19 mRNA vaccines. These vaccines, such as the Pfizer-BioNTech and Moderna vaccines, instruct cells to produce a harmless spike protein found on the surface of the SARS-CoV-2 virus, the pathogen responsible for COVID-19. Due to this novel technology, we saw an unprecedented acceleration in vaccine development and production, leading to the successful immunization of millions of individuals worldwide within a remarkably short timeframe [5]. However, it is important to note that this remarkable success has not been mirrored in the field of mucosal vaccines, where a notable disparity in progress persists. Mucosal vaccines, which offer a distinct approach to immune defense, have not yet realized their full potential, especially when considering the nasal route of administration. Several factors contribute to this divergence, including the complex and dynamic nature of the nasal mucosal environment. Despite the substantial progress in parenteral vaccine technology, addressing the complexities inherent to mucosal vaccine development remains a critical frontier in our ongoing battle against infectious diseases.

When exploring mucosal vaccines and their potential to transform immune responses at mucosal sites, it becomes evident that their effectiveness depends on precise targeting. At the center of our immune defense system lies a crucial group of molecules known as pattern recognition receptors (PRRs). These specialized receptors are found on the surface of various immune cells, including APCs such as DCs and macrophages. PRRs serve as the sentinels of our immune system, constantly scanning their environment for molecular signatures indicative of invading pathogens. Among these receptors, C-type lectin receptors (CLRs) stand out as key contributors to the orchestration of immune responses. CLRs are crucial endocytic receptors with a unique capacity to recognize and internalize pathogen-specific carbohydrate structures. This exceptional attribute makes CLRs prime targets for mucosal vaccine development.

The present review provides an overview of the nasal structure and its immune-related environment, with particular emphasis on the benefits and obstacles associated with the delivery of vaccines through the nasal route. The author’s focus was directed toward next-generation approaches intended to improve the nasal route for administering antigens, specifically by targeting CLRs, such as the mannose receptor and macrophage galactose-type lectin (MGL) receptor. Furthermore, while not aiming to provide an exhaustive review of targeted glycoconjugate vaccines, we intend to enhance our understanding by discussing the key developments in this field and highlighting recent advances in the application of lactobionic acid and mannose as components that target APCs in nasal vaccine delivery.

## 2. Mucosal Vaccines: Prospects and Obstacles

Given that a significant proportion of invading infectious agents enters the host organism through mucosal surfaces, mucosal vaccines have garnered heightened interest as a promising alternative to injectable vaccines. Unlike injectable vaccines, mucosal vaccines have the potential to elicit mucosal immunity, providing defense against pathogenic infections while also triggering a robust systemic immune response [6]. Nevertheless, the number of approved mucosal vaccines remains quite limited. At present, fifteen mucosal vaccines have been approved for human use: ten administered orally, four through the intranasal route, and one using a nebulizer to convert a liquid vaccine into an aerosol for inhalation through the mouth. Most of these vaccines are based on formulations exploiting either live attenuated viruses or whole-cell inactivated components. Additionally, some vaccines approved for mucosal use contain non-replicating viral vectors as their platform (Table 1) [7,8,9]. Table 1 highlights the key features and fundamental concepts of these vaccines. Although these types of immunizations have significantly improved illness prevention, it is worth noting that certain vaccines, like Vivotif, might exhibit differing degrees of response effectiveness, depending on the prevailing strain at the time.

Live attenuated vaccines, whole-cell inactivated vaccines, and non-replicating viral vector vaccines exhibit high efficacy in stimulating humoral and cell-mediated immune responses that persist for long periods of time. Nonetheless, these vaccines have the potential to trigger allergic reactions, autoimmune responses, and excessive inflammation. There is also a concern that the attenuated pathogens employed in vaccine manufacturing could revert to their virulent states [41].

Non-replicating viral vectors, although safer than live attenuated viruses, may elicit immune responses against the viral vector itself, particularly against its capsid proteins, potentially reducing efficacy upon repeated dosing [42]. To address these concerns, current mucosal vaccine research is increasingly focusing on safer alternatives, such as subunit vaccines and those based on nucleic acids [43]. Due to their composition of minimal pathogen segments, these vaccines present fewer risks, however their immunogenicity is comparatively diminished. Consequently, they are consistently administered alongside an adjuvant (immune enhancer) and/or a specialized delivery system [44]. The scarcity of available mucosal vaccines can be attributed to the absence of efficient delivery systems capable of maintaining the structural integrity of vaccine antigens and potent adjuvant effects [45]. This challenge is further deepened by the inherent tendency of the mucosal immune system to induce a state of tolerance [46] due to the substantial presence of antigens and microorganisms, which requires essential mucosal immunoregulatory mechanisms. These mechanisms play a crucial role in upholding homeostasis and minimizing the development of harmful chronic inflammatory reactions. Additionally, we must not overlook the fact that the mucosal surface has a robust defense mechanism that includes physical, chemical, and biological barriers that effectively prevent the intrusion of pathogens. Tightly interconnected epithelial cell junctions, the continuous movement of cilia, and the mucus secretion orchestrated by goblet cells and seromucous glands within mucosal tissue represent the majority of the physical barrier’s features [47]. Approximately 95% of the mucus composition comprises water, while mucin makes up around 2.5 to 3%. The remaining 2% encompasses a blend of electrolytes, enzymes, proteins, antibodies, lipids, and cellular debris. These mucin glycoproteins are crucial in giving mucus its characteristics of viscosity and a gel-like consistency. The type of mucosal surface affects the pH value of the mucus and the density of the mucus layer. Notably, the mucus in the lungs and nasal cavity typically exhibits a pH that is close to neutral [48]. The chemical defense encompasses factors like antibacterial proteases (exopeptidases and endopeptidases), an array of digestive enzymes, lysozymes, distinct pH conditions, and mucopolysaccharides. Regarding the nasal mucosa, the enzymatic degradation processes are significantly influenced by the cytochrome-P450-dependent monooxygenase system [49]. The cytochrome P450 is the main family of phase I enzymes that promotes the oxidative biotransformation of a majority of drugs and various lipophilic xenobiotics [50]. Concerning the biological barrier, this is constituted by the presence of microbiomes (intestinal flora and genital tract flora) along with their resultant metabolites. For example, researchers have illustrated how the commensal protist *Trichomonas musculis*, found in mice, acts as a defense against enteric bacterial infections. This protective mechanism involves triggering inflammasome signaling, subsequently fostering Th1 and Th17 immune responses orchestrated by DCs. Yet, it is important to note that these protective barriers can also hamper the entrance of conventional vaccines into the organism [51].

However, on a more encouraging note, mucosal vaccines offer the flexibility of administration through a variety of routes, each triggering distinct types of immune responses. The benefits of employing mucosal administration for vaccines include the induction of dual immunity at both mucosal and serosal sites, as well as the generation of immune responses on both local and distant mucosal surfaces [52]. This is supported by a defining attribute of the mucosal immune system, which is the presence of specialized lymphoid tissue known as mucosa-associated lymphoid tissue (MALT). MALT includes structures that are designated as nasopharynx-associated lymphoid tissue (NALT), bronchus-associated lymphoid tissue (BALT), and gut-associated lymphoid tissue (GALT), based on their specific locations [53]. Moreover, mucosal vaccines present numerous benefits in contrast to injectable vaccines. These advantages encompass simplified administration, enhanced patient compliance, reduced expenses, elimination of needle-related injuries and disposal concerns, and the potential for large-scale immunization efforts [54].

In summary, while nasal mucosal vaccination faces challenges related to antigen delivery, limited dosing volume, restricted membrane permeability for hydrophilic compounds, the action of mucociliary clearance, the presence of the mucus barrier, and the enzymatic milieu within the nasal mucosa [55], it also offers opportunities for enhanced immune responses, mucosal homing, ease of administration, and targeted immunity. Overcoming these challenges and harnessing the opportunities could lead to the development of effective and innovative vaccines for a wide range of infectious diseases.

## 3. Distinctive Elements of Mucosal Immune Response: An Introduction to Nasal Vaccines

The selection of distinct pathways in the mucosal immune system significantly influences the identification of immune response sites, the type of immune response generated, and the patterns of lymphocyte migration [56]. These factors notably impact the effectiveness of vaccines. Consequently, the appropriate mucosal routes are chosen based on an assessment of the characteristics of the viral infection. For instance, oral immunization typically triggers immune responses within the gastrointestinal tract, alongside the oral mucosa and mammary glands [57]. On the other hand, intranasal administration effectively prompts antibody production in salivary glands, nasal-associated lymphoid tissue, bronchus-associated lymphoid tissue in the lower respiratory tract, as well as the urogenital tract [58]. The specificity of mucosal immune stimulation through the intranasal route is influenced by distinct markers of B cells generated at the inductive site. B cells activated within NALT display unique qualities, including the presence of α4β1-integrin and CCR10 receptors, enabling them to effectively migrate toward regions like the respiratory and genitourinary tracts, which are regions that express high levels of their corresponding ligands VACM-1 and CCL28 [59]. Therefore, the choice of immunization route should be thoughtfully considered, depending on the specific mucosal sites targeted by different pathogens.

Among the various routes of mucosal administration, intranasal delivery sets itself apart as particularly favorable and well-received. This preference is due to the fact that the nasal delivery pathway circumvents the hepatic first-pass effect and is more favorable for antigens than the oral route, thereby the antigen dosage can be diminished [60]. Research has demonstrated that vaccination through the nasal route leads to a quicker initiation of immune responses and the establishment of long-lasting immune memory compared to oral immunization. This phenomenon can be attributed to the abundant population of DCs and macrophages within NALT.

### 3.1. Immunological Significance of the Nasal Mucosa

The nasal passage plays a significant role in the mucosal immunity of the upper airways, contributing to both protection of the host and maintaining a balance between the commensal microbiota and potential pathogens. This dual role includes immune protection and harmonious interaction within this critical region [61]. During the early 2000s, an important turning point occurred with the approval in the United States and Canada (FluMist) of the first nasal vaccine against the influenza virus for human use. The efficacy of this vaccine has contributed to the growth of the NALT research community, further enhancing our fundamental understanding of immune responses in the nasal region [62].

The physiological architecture of the nasal cavity is a crucial aspect in the development of nasal vaccines. This cavity is anatomically divided into distinct compartments, namely the vestibular, respiratory, and olfactory regions. The anterior portion of the nasal cavity is constituted by a stratified squamous epithelium, which transitions to a pseudostratified columnar ciliated epithelium in the posterior portion. This unique epithelial structure consists of various cell types, including non-ciliated and ciliated columnar cells, basal cells (involved in regeneration and differentiation), and goblet cells responsible for mucus secretion [63]. The respiratory region comprises mucus-producing cells, numerous blood vessels, and the respiratory epithelium, being the primary route via which the vaccine gains access into the human body. Also responsible for humidifying, filtering, protecting, and removing particulate matter, it is considered the main part within the nasal cavity. Furthermore, the majority of respiratory epithelial cells is covered with cilia, which are microscopic projections that serve an important function: the mechanical inhibition of the entrance of foreign substances, including pathogens [64]. A distinctive attribute of the nasal cavity is its physical proximity to the central nervous system. This anatomical location contains olfactory receptors within the upper region of the nasal cavity. These receptors consist of specialized nerve cells responsible for detecting odors [65]. As a result, it is essential to consider the potential passage of antigens to the nervous system through the olfactory nerve when developing nasal vaccines. Therefore, the careful design of the antigen carrier is crucial, keeping in mind factors such as particle size, surface modifications to target receptors other than olfactory neurons, and the lipophilicity of the formulation, all of which can impact the likelihood of crossing into the nervous system [66].

### 3.2. The Immune System Specific to the Nasal Cavity’s Mucosal Region

In humans, nasal lymphoid tissue is located in the oropharynx that forms a circular tonsillar ring, referred to as Waldeyer’s ring. This ensemble includes nasopharyngeal adenoids (or tonsils), as well as paired tubal tonsils, palatine tonsils, and lingual tonsils [67]. NALT harbors a diverse range of immune cells essential for instigating antigen-specific immune reactions. These include B and T cells, along with APCs like DCs and macrophages. NALT is also surrounded by an epithelial layer housing specialized microfold (M) cells, which play a crucial role in the uptake of antigens, primarily in the particulate form through phagocytosis and transcytosis across epithelium [68]. This phenomenon occurs due to the absence of a brush border in villous M cells, which makes it easier for antigens to bind and be delivered. These cells are also unable to secrete mucus or any enzymes. In addition, it is important to point out that M cells should not be regarded as typical antigen-presenting cells. Instead, they serve as “antigen transporting cells”, responsible for conveying luminal particles and antigens to conventional DCs for subsequent antigen presentation [69]. For soluble antigens, uptake occurs passively through absorption from the epithelial membranes. Meanwhile, APCs, such as DCs and macrophages, can also play an active role in acquiring particulate antigens at the nasal mucosa, then traveling to the lymph nodes. DCs have the ability to disrupt the tight junctions among epithelial cells, extending dendrites beyond the epithelial layer to directly access and sample pathogens. To ensure both the protection of the epithelial barrier’s integrity and the ability to breach well-established tight junctions, DCs express proteins associated with these tight junctions [70]. DCs become activated and mature when they come into contact with antigens. The nature and dosage of adjuvants administered alongside the antigen play a crucial role in determining this activation. These matured cells subsequently migrate to the interfollicular T-cell zone, initiating the activation of naive T cells. Eventually, effector T cells transition toward the germinal centers within B-cell follicles, where they release cytokines that facilitate the process of IgA class-switch recombination [71]. These mucosal T and B lymphocytes that are stimulated are able to pass into the blood’s circulation. Subsequently, they might return to the initial mucosal sites of activation or migrate to other mucosal regions distinct from their priming sites, thereby establishing an interconnected network within the mucosal immune system, referred to as the common network of the mucosal immune system [72].

IgA manifests in a variety of molecular structures, which sets it apart from other immunoglobulin classes. While monomeric IgA predominates in human serum, the prevailing form of IgA at mucosal linings is dimeric [73]. Dimeric IgA consists of a pairing of two IgA monomers. Each monomer is composed of two heavy and light chains, and this arrangement is strengthened through the presence of disulfide bridges and the inclusion of the joining (J) chain [74]. Within the mucosal effector site, dimeric IgA, produced by the local plasma cells, interacts with the polymeric Ig receptor (pIgR), crossing the epithelial barrier via transcytosis. Subsequently, proteolytic cleavage facilitates their release into the luminal space, leading to their transformation into secretory IgA (sIgA) [75]. In addition to its other roles, the secretory components within sIgA offer protection against degradation by proteolytic enzymes. IgA is strengthened in order to withstand the difficulties presented by the rigorous mucosal milieu [76]. Hence, this immunoglobulin is of extreme significance as a mucosal immune effector. Its functionality encompasses three essential processes: expelling antigens by having sIgA attach to them and subsequently relocating the antigens from the lamina propria, establishing immune exclusion, and neutralizing intracellular antigens [77]. The neutralization of the cholera toxin is an example of immune exclusion; in this process, sIgA entraps antigens in the mucus, preventing their interaction with cell-surface receptors and their subsequent effects. Moreover, depending on the kind of antigen, IgA synthesis may take place through T-independent or T-dependent pathways. High-affinity IgA antibodies are developed in a T cell-dependent manner, whereas IgA generated via T-independent routes exhibits reduced or inadequate antigen specificity due to restricted somatic hypermutation [78]. The latter situation results in low-affinity antibodies that are typically linked to immune reactions targeting antigens originating from the resident microbiota [79]. However, it is important to note that this classification is somewhat oversimplified since, in reality, a majority of pathogens exhibits a combination of T-cell-dependent and -independent mechanisms. This complexity results in hybrid immune responses, where certain T-independent epitopes might trigger T-dependent reactions. Nevertheless, the field of subunit vaccine production is a critical area where this differential is significant, even clinically. In such instances, polysaccharides are occasionally attached to carbohydrates or proteins, supporting the development of robust T-dependent responses, which is particularly important for therapeutic vaccines [80].

### 3.3. Key Aspects of Immune Response Mechanisms in Vaccinations

When compared to the systemic immune system, the mucosal immune system is distinctly characterized by differences in antigen presentation, effector roles, and cellular composition [81]. The mucosal immune system can be categorized into two main components: inductive sites, where antigens gathered from mucosal surfaces activate naive T and B lymphocytes, and effector sites, where effector cells, having undergone extravasation, retention, and differentiation, execute their functions, eliciting a specific immunological response [82]. Given the significant role that APCs play in coordinating immune reactions within mucosal regions, it becomes essential to characterize the professional APCs, like DCs and macrophages, which exist in the nasal mucosa, elucidating their distinct subtypes and respective functions.

DCs are central to orchestrating both innate and adaptive immunity. These cells undergo two distinct functional phases: immature and mature. In their resting state, DCs exhibit an immature profile, characterized by these cells’ ability to capture antigens directly from their surrounding environment. This process is facilitated by a specialized endocytic system, which involves numerous uptake receptors [83]. The processes of activation and maturation occur when DCs are exposed to “a triggering signal”, involving a complex series of modifications in both their characteristics and functions [84]. Maturation dictates the processing of antigens by acidifying endocytic vacuoles, triggering proteolysis, and transferring peptide-major histocompatibility complexes (MHCs) to the cell surface. The antigens/proteins are typically processed into peptides via two pathways: the endogenous route, where cytosolic proteins are directly degraded by the proteasome and the resulting peptides are transported into the endoplasmic reticulum with the assistance of the transporter associated with antigen processing (TAP) proteins for presentation on MHC class I molecules to CD8^+^ T cells; or the exogenous route, where peptides become entrapped in early endosomes, gradually progressing through the vacuolar system, and undergoing lysosomal protease degradation for presentation on MHC class II molecules to CD4^+^ T cells [85]. Nonetheless, DCs possess the ability to cross-present external antigens to CD8^+^ T cells, a phenomenon known as cross-presentation [86]. Interestingly, the administration of DNA vaccines eliminates the necessity for cross-presentation. This is due to the fact that antigens encoded by DNA vaccines are internally generated and follow the conventional MHC class I pathway [87].

Mature DCs undergo surface remodeling, which reduces their capacity for phagocytosis. They frequently display numerous costimulatory markers connected to the B7 (CD80 and CD86), the TNF (CD40), and the Notch families, along with soluble factors like type I interferon and IL-12 [88]. Besides these costimulatory molecules, the enhancement of MHC classes I and II, adhesion molecules and chemokine receptors are vital for the DCs’ migration to the lymphoid tissues, like the lymph nodes, and for creating steady and enduring interactions with T cells within the immunological synapse. The differentiation of T cells into memory or effector phenotypes depends on this interaction, which, in turn, might be of an inhibitory or stimulatory nature [89]. This phenomenon can be explained by the fact that the same ligand, expressed on the cell surface of naïve T cells can bind to either co-stimulatory or co-inhibitory receptors within the APC. For example, CD80 and CD86 have the capacity to bind with either CD28 or CTLA-4, resulting in either a favorable or suppressive T-cell response, respectively [90].

Likewise, in order to develop a more comprehensive understanding of the intricate process of DC migration, it is imperative to understand the interaction of chemokine receptors and integrins. This interplay not only guides the activated, mature DCs toward lymph nodes, but also facilitates their exit from tissue environments. Specifically, there is an increase in the presence of CCR7 and CXCR4 expressions, while the activity of the α5β1 integrin and the chemokine receptor CCR6, the latter being correlated with the migration of immature DCs to sites of inflammation, is reduced [91]. Subsequently, the CCR7^+^ DCs undergo migration, moving deliberately toward areas with greater concentrations of CCL21, which is the corresponding functional ligand of CCR7, in a process called chemotaxis [92].

Within the immunological synapse, DCs play a critical role in T-cell proliferation and differentiation, leading to the development of diverse subsets of T helper cells. This differentiation relies on lineage-specific cytokines and the activation of transcription factors [93]. For instance, in the presence of the transcription factor Tbet, the signal transducer and activator of transcription 4 (Stat4) and the cytokines IL-12 plus IFN-ɤ, the naïve CD4^+^ T cells polarize to a Th1 phenotype, associated with cellular immune response. Conversely, in the presence of the transcription factor GATA-3 and the cytokines IL-4 plus IL-2, the naïve CD4^+^ T cells polarize to a Th2 phenotype, associated with the activation of B cells and the production of antibodies [94]. The polarization to a Treg phenotype involves the transcription factor FoxP3 and the cytokines TGF-β plus IL-2, whereas the Th17 phenotype encompasses the transcription factor RORγt, the signal transducer and activator of transcription 3 (Stat3), and the soluble factors TGF-β plus IL-6, IL-21, and IL-23 [95]. Additionally, recent insights reveal that T cells in the immunological synapse release extracellular vesicles (EVs), contributing to the modulation of immune response through intercellular communication [96]. The functions of DCs are influenced by these EVs via their surface proteins and enclosed cargo. These contents may encompass co-stimulatory molecules (such as CD2 and CD28), integrins (like lymphocyte function-associated antigen-1, LFA-1), microRNAs, the T-cell antigen receptor complex, and cytokines [97].

**Figure 1:**
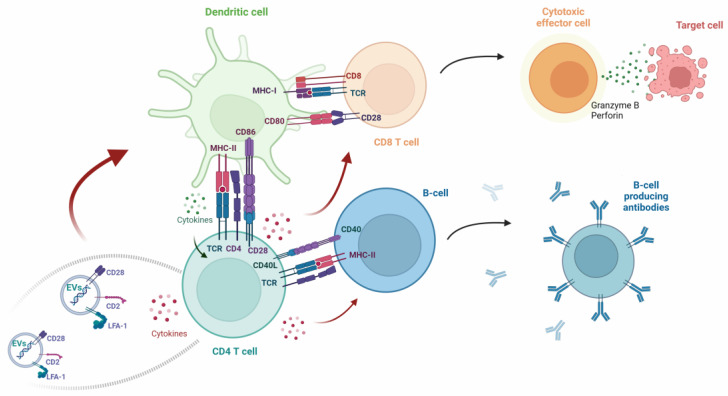
Schematic illustration of the immune response generated after antigen presentation, highlighting key interactions and communication between immune cells. The mature DC interacts with T cells through both MHC class I and class II molecules, presenting antigens to CD8^+^ and CD4^+^ T cells, respectively. This interaction leads to T-cell activation. Activated CD4^+^ T cells engage with B cells through CD40-CD40L interactions, providing essential co-stimulatory signals. This interaction leads to B-cell activation and antibody production. Activated CD8^+^ T cells secrete perforins and granzymes that attack target cells, contributing to the elimination of infected or abnormal cells. The T cells can also release EVs containing various signaling molecules, including integrins, cytokines, and microRNAs. These EVs can travel to nearby DCs and other immune cells, where they can influence gene expression, cytokine production, and antigen presentation, facilitating immune cell communication and modulation. Created with BioRender.com.

In summary, the functions of DCs are intricately intertwined with the orchestration of immune responses, involving a subtle interplay between antigen presentation, expression of co-stimulatory or co-inhibitory molecules, cytokine signaling, chemotaxis, and intercellular communication through EVs, ultimately shaping the immune response in a finely tuned manner (Figure 1).

### 3.4. APCs in Human Nasal Mucosal Tissues

In the intricate environment of the human nasal mucosa, two critical players stand out: macrophages and DCs. These immune surveillance cells are not mere onlookers; they orchestrate the immune response in this specialized setting, protecting against invading pathogens and preserving the difficult balance between tolerance and defense.

#### 3.4.1. Macrophages

Macrophages are abundant in many tissues, acting as specialized phagocytes of the immune system. For many years, it was believed that macrophages constitute a reasonably uniform group of mononuclear phagocytes originating from hematopoietic stem cells in the bone marrow [98]. Nonetheless, the emergence of innovative fate-mapping models, coupled with the utilization of systems biology strategies, revealed that a significant portion of tissue-resident macrophages is derived from embryonic precursors that colonized tissues during fetal development [99]. They maintain their populations through local self-renewal without the need for continuous recruitment from the bloodstream [100]. The heterogenicity among tissue-resident macrophages is an essential outcome of their distinct functions within specific tissues and microanatomical niches during both developmental stages and adulthood. This diversity is crucial for maintaining tissue functionality and equilibrium [101]. However, unlike DCs that communicate information from peripheral sites to lymph nodes, tissue-resident macrophages seem to execute their roles directly within their local environment [102].

In the context of the nasal mucosa, macrophages exhibit strategic localization, frequently gathering in specialized areas, such as the lamina propria and submucosal layer [103]. As a result of their close proximity to blood vessels and the epithelium, macrophages are in a position to respond quickly to pathogens or other danger signals, which helps to support the body’s overall immune defense and maintain tissue integrity in the nasal mucosal environment. Macrophages possess the ability to eliminate pathogens, initiate inflammatory responses, and engulf dead cells. This arises from the fact that, as sentinels cells, they express diverse surface receptors, encompassing scavenger, complement, Fc, and other pattern recognition receptors (such as TLRs, CLRs, NLRs, and RLRs) [104]. Furthermore, macrophages possess a continuous expression of MHC II molecules, facilitating their efficient participation in the presentation of antigens to CD4^+^ T lymphocytes. This interaction and the macrophages’ ability to secrete cytokines that affect the differentiation and polarization of T cells and chemokines that attract other immune cells to the site of infection lead to the initiation of robust inflammatory adaptive responses [105]. Moreover, macrophages work closely with other immune cells, including B cells and DCs, to shape and regulate the immunological environment. Collectively, they influence DCs’ maturation process and transmit antigenic information, which facilitates the DCs’ antigen-presenting capacity and their subsequent interaction with T cells. Macrophages also support B-cell proliferation and antibody class switching via antigen presentation and by releasing molecules as a B-cell activating factor (BAFF) [106]. Moreover, Zhou and colleagues reported that macrophages have the ability to express CD154 (also known as CD40L), a constituent of the TNF family. This protein expression enables the interaction with CD40 molecules present on B cells, subsequently fostering their activation and survival and participating in the regulation of antibody generation [107].

The capacity of macrophages to adapt and change, phenotypically and functionally, in response to certain local signals is one of their most distinguishing characteristics. Macrophages adopt distinct phenotypes that may be identified in terms of gene expression, surface molecule patterns, and the synthesis of biological mediators and metabolites in order to carry out these various immunological activities [108]. Generally speaking, macrophages adopt distinct activation states, commonly referred to as M1 (classical) and M2 (alternative) phenotypes. These distinct profiles are associated with immune responses, contributing to fine-tuning the inflammatory response [109].

M1 macrophages are characterized by their pro-inflammatory properties, ideal for fighting infections and rapidly responding to threats. These cells are activated by the granulocyte–macrophage colony-stimulating factor (GM-CSF) [110], interferon-gamma (IFN-γ), lipopolysaccharide (LPS), and tumor necrosis factor-alpha (TNF-α). Upon activation, they release several pro-inflammatory cytokines, including TNF-α, interleukin-6 (IL-6), IL-23, IL-12, and IL-1β [111,112]. Additionally, they generate reactive oxygen species (ROS) and reactive nitrogen species (RNS) and increase the expression of costimulatory markers, such as CD80 and CD86, as well as surface markers CD64 and CD68 [113]. They also upregulate enzymes like indoleamine 2,3-dioxygenase (IDO) and inducible nitric oxide synthase (iNOS) [114]. Furthermore, M1-like macrophages secrete matrix metalloproteinases (MMPs), enzymes that efficiently break down components of the extracellular matrix, enhancing the clearance of pathogens from the tissues [115]. M1 macrophages also recruit immune cells by expressing M1-derived chemokines like CCL5, CXCL9, CXCL10, and CXCL5, and initiate robust immune reactions against pathogenic invaders [116]. On the other hand, M2 macrophages are associated with anti-inflammatory and tissue repair functions. They are crucial for dampening excessive inflammation, fostering tissue healing, and boosting immune tolerance in order to prevent collateral damage to susceptible mucosal tissues [117]. These M2 macrophages can be further divided into four distinct subgroups: M2a (after IL-4 and IL-13 stimulation), M2b (triggered by immune complexes combined with TLRs and/or IL-1 receptor ligands), M2c (elicited by IL-10, TGF-β, or glucocorticoid hormones), and M2d or tumor-associated macrophages (induced by TLR or adenosine receptor agonists) [118]. Surface markers like CD163, CD206, and CD209/DC-SIGN along with the expression of the arginase enzyme and the presence of anti-inflammatory cytokines, such as IL-10 and TGF-β, are notable features of M2 macrophages. Among M2 macrophages, the M2a subtype is further characterized by the expression of chemokines such as CCL17, CCL22, and CCL24. These chemokines facilitate the recruitment of Th2 cells, regulatory T cells, eosinophils, and basophils. Importantly, these macrophages exhibit enhanced phagocytic capacity, characterized by the presence of receptors, such as Dectin-1 and DCIR, and are closely associated with tissue repair and cell growth processes [119]. YM1, chitinase-like protein 3, is crucial for M2a macrophages due to its active role in tissue remodeling and repair, promoting the resolution of inflammation and facilitating the healing of damaged tissues [120]. M2b macrophages, also called regulatory macrophages, secrete cytokines like IL-10, IL-1, IL-6, and TNF-α, as well as significant amounts of the chemokine CCL1. This orchestrated release of molecules serves to intricately modulate the intensity of inflammatory responses [121]. M2c macrophages exhibit elevated levels of arginase along with the chemokines CCL16, which contributes to the recruitment of eosinophils, and CCL18. Additionally, these macrophages are instrumental in supporting processes such as angiogenesis, tissue regeneration, and the phagocytosis of apoptotic cells [122]. Particularly, for the last process, the contribution of myeloid–epithelial reproductive tyrosine kinase (MerTK) is critical. MerTK functions as a receptor that specifically binds to phospholipids exposed on the surface of apoptotic cells, which are negatively charged [123]. M2d macrophages are primarily recognized for their elevated levels of IL-10, vascular endothelial growth factors (VEGFs), and TGF-β. They also secrete chemokines CXCL10, CXCL16, and CCL5, while their production of IL-12 and TNF-α cytokines is notably diminished. These macrophages play a significant role as the predominant inflammatory element within tumoral tissues, actively influencing processes such as angiogenesis and the facilitation of tumor metastasis [124]. In summary, the presence of both M1 and M2 macrophages in the human nasal mucosal environment ensures a balanced and effective immune response. This dynamic interplay between M1 and M2 macrophages is essential for maintaining the health and integrity of nasal mucosa in the face of various challenges (Table 2).

Recognized in recent years as part of the macrophage family [125], Langerhans cells represent a unique subset of mononuclear phagocytic cells, typically confined to the epidermis and closely intertwined with keratinocytes [126]. This cell population exhibits evolutionary conservation and can ensure their cellular network by self-renewal [127]. Although LCs descend from the same developmental origin as other tissue-resident macrophages, their functional characteristics closely resemble those of DCs. This similarity is evident in their capability to migrate and activate T cells [128]. LCs are distinctly recognizable as the sole cells within the epidermis that, when activated, exhibit an increase in MHC II expression. These MHC II^high^ LCs are then capable of extending vertically their dendrites through tight junctions. Conversely, this phenomenon is absent in non-activated LCs, indicating the specialized nature of these activated LCs in antigen acquisition. Particularly in conditions where skin barriers are compromised, LCs possess the ability to initiate T-cell-mediated immune responses [129]. This is especially evident when the yeast *Candida albicans* is recognized, in which LCs induce the activation of Th17 cells. Nonetheless, LCs exhibit remarkable functional plasticity, and the type of immune response they elicit appears to rely on the prevailing immunological circumstances, as these cells have also been observed to initiate immune tolerance in certain situations [130]. The differentiation process of LCs relies on essential factors like TGF-β (specifically bone morphogenetic protein-7 (BMP-7)), Axl, and NOTCH signaling [131]. Furthermore, LCs also exhibit the presence of the C-type lectin receptor identified as Langerin, a factor contributing to the formation of Birbeck’s granules. This phenomenon arises due to the continuous internalization of the receptor, leading to its accumulation within endosomal compartments [132]. LCs can additionally be identified through the presence of markers such as the epithelial cell adhesion molecule (EpCam), also known as CD326, and CD1a [133].

#### 3.4.2. Dendritic Cells

Precursor cells in the bone marrow are the source of DCs, which can be divided into different subsets based on differences in their origin (myeloid lineage or lymphoid lineage), migratory behaviors (migratory or tissue-resident DCs), growth factor dependence, and specialized roles in the immune system (immunogenic or tolerogenic DCs) [134]. As a result, DCs are categorized into conventional DCs (cDCs), commonly referred to as classical or myeloid DCs, plasmacytoid DCs (pDCs), and monocyte-derived DCs (MoDCs). There is evidence that steady-state cDCs and pDCs originate from common DC progenitors, being functionally dependent on the cytokine receptor Flt3 (CD135), unlike MoDCs [135].

At first known as inflammatory DCs, MoDCs emerge from precursor monocytes, encompassing both the classical CD14^+^ monocytes and the non-classical CD16^+^ monocytes [136]. When these monocytes infiltrate the airway mucosa as part of an inflammatory reaction, they have the capacity to enhance the expression of MHC class II glycoproteins, as well as the integrins CD11c and CD11b, leading to their differentiation into MoDCs. This expression pattern makes it difficult to distinguish from cDCs. Nonetheless, a characteristic feature of their monocyte lineage is the expression of CD64 (also known as FcγRI) [137]. Similar to classical monocytes, MoDCs rely on the cytokine macrophage colony-stimulating factor (M-CSF) and the C-C motif chemokine receptor 2 (CCR2) to be recruited to sites of inflammation. Their usual roles within tissues encompass activities like presenting antigens to effector T cells and generating cytokines as TNF-α and the production of nitric oxide synthase (iNOS) [138].

pDCs play a significant role in generating type I and III interferons as a response to viral infections involving double-stranded DNA and single-stranded RNA, activating its receptors TLR9 and TLR7, respectively. Both DC maturation and CD8^+^ T-cell activation, which are essential mediators of antiviral immunity, depend on this function [139]. pDCs are primarily found in the T-cell regions within lymph nodes and exhibit minimal expression of extracellular toll-like receptors (TLRs), which contributes to their limited ability to present antigens [140]. Consequently, they are associated with inducing tolerance when robust inflammatory signals are absent [141]. pDCs are devoid of myeloid markers like CD11c, as well as lineage markers CD3, CD19, CD56, CD14, and CD16. However, they do exhibit the presence of various distinctive gene products, including blood dendritic cell antigen-2 (also recognized as CD303), blood dendritic cell antigen-4 (also termed CD304), CD123, and CD45RA [142].

Conversely, cDCs express myeloid markers (CD11c) and stand out as the principal antigen-presenting cells for T lymphocytes. These cells predominantly reside in the marginal zone of lymph nodes and display the presence of both extracellular and endosomal TLRs. cDCs have been additionally categorized into two distinct subsets based on their levels of CD141 expression (also identified as BDCA3 and thrombomodulin, or cDC1s) and CD1c expression (also referred to as BDCA1 or cDC2s) [143]. Each subset of cDCs exhibits unique gene expression patterns, which point to specific and specialized roles. A special feature of the CD141^+^ DCs subpopulation is the presence of DNGR-1 (CLEC9A), a CLR. DNGR-1 detects polymeric F-actin that becomes exposed during cell death by necrosis. Then, this receptor activates its signaling pathways, namely through spleen tyrosine kinase (SYK), facilitating the cross-presentation of antigens associated with tissue damage to CD8^+^ T cells [144]. Regarding TLRs, CD141^+^ DCs demonstrate the presence of TLR3, TLR8, and TLR9, while being devoid of TLR4, TLR5, and TLR7 [145]. Furthermore, the development of CD141^+^ DCs requires the presence of essential transcription factors such as IRF8, ID2, and BATF3. These cells play a crucial role in coordinating immune responses against intracellular pathogens, leading to the secretion of substantial amounts of IL-12p70 and type III interferons [146,147]. They are instrumental in triggering T helper 1 (Th1) and cytotoxic T-cell responses through an engagement with the MHC class I pathway [148]. The expressions of the adhesion protein nectin-like molecule 2 (Necl2/CADM1) and X-C motif chemokine receptor 1 (XCR1), both of which play important roles in activating CD8^+^ T lymphocytes, are distinguishing characteristics of these responses [149]. The CD1c^+^ DC subset possesses the ability to present antigens to both CD4^+^ and CD8^+^ T cells, although they are more proficient at doing so for CD4^+^ T cells via the MHC II pathway [150]. CD1c^+^ DCs play a role in stimulating immune reactions targeted at extracellular bacteria and fungi, due to their capacity to generate Th2 and Th17 responses. However, under appropriate stimulation, these cells can also produce substantial levels of IL-12, promoting the induction of Th1 and cytotoxic immune responses [151]. Despite emerging from common DC progenitors, CD1c^+^ DCs exhibit distinct characteristics from MoDCs in terms of phenotype and functionality. However, the overlap in marker expression, such as CD14, which can be shared by both cell types, highlights the need for additional markers to more accurately distinguish DC populations [152]. Investigations into human CD1c^+^ DCs have revealed that this population comprises cellular subsets with distinct surface expressions of CD5. The expression of CD5 was assessed in CD1c^+^ myeloid DCs in the blood and bone marrow, and its association was observed in certain myeloid malignancies, including acute myeloid leukemia and chronic myelomonocytic leukemia [153]. As a result, researchers have put forth the idea of distinct subgroups within the CD1c^+^ DCs subset. As an example, Yin and colleagues introduced two additional subpopulations based on the expression level of CD5. Their findings revealed that, in response to R848, CD5^high^ cDC2s generated IL-23. In contrast, the CD5^low^ population responded to the same TLR7/8 agonist by producing low levels of IL-23 and secreting IL-6, TNF-α, IL-12, and moderate levels of IFN-β [154]. Moreover, the CD1c^+^ DCs subset can be recognized through the presence of CD172a/SIRPα and CLEC10A (also recognized as MGL or CD301) markers, which are also employed to delineate other cells [83]. For instance, monocytes and granulocytes both express CD172a and monocytes grown with GM-CSF and IL-4 in vitro also express CD301, as well as macrophages. IRF4 is a transcription factor essential for the development of CD1c^+^ DCs, while Notch signaling, particularly Notch2, plays a role depending on the subset of CD1c^+^ DCs [155]. These cells also display the presence of CLRs like the DC immunoreceptor (DCIR or CLEC4A), dectin-1 (CLEC7A), dectin-2 (CLEC6A), DEC-205 (CD205), and the mannose receptor (CD206) [156,157]. In humans, mutual activation between cDCs and pDCs has been observed upon appropriate stimulation, showcasing their ability to collaborate and yield synergistic effects [158].

After providing an overview of the many DC subsets present in various physiological situations (Table 3), it is now pertinent to focus specifically on the unique array of DC subsets present in the complex environment of the human nasal mucosa. A sophisticated analysis of the DC subsets in the nasal mucosa reveals the existence of CD207^+^ Langerhans cells and plasmacytoid DCs. Notably, these pDCs were identified in substantially higher amounts than in the oral mucosa [159]. These two cell types collaborate synergistically, fulfilling distinct functions in the nasal environment. LCs are primarily engaged in antigen presentation and the initiation of immune responses, whereas pDCs excel in providing antiviral protection and modulating immune activity [160].

Concerning the type of myeloid cells found within both NALT and non-NALT tissues, researchers identified murine cDC1s by the co-expression of CD103 and EpCam, while lacking CD11b expression. In contrast, murine cDC2s are consistently positive for CD11b and can be further categorized based on their expression of CD103 and EpCam. Notably, not all cDC2s express both markers; different combinations of CD103 and EpCam may be present, with some cells lacking one or both markers, depending on the tissue context [161]. This implies potential functional heterogeneity within this DC population. For instance, EpCam typically participates in cell–cell adhesion and interactions, potentially playing a role in upholding the immunological synapse between APCs and T cells [162]. Meanwhile, CD103 is linked to mucosal locations and contributes to mucosal T-cell homing induction and the preservation of mucosal tolerance [163]. Nonetheless, these markers (CD103 and EpCam) are generally not used for characterizing DCs in human nasal tissue. Instead, human CD141^+^ (cDC1) DCs are identified by the marker BDCA-3, while human CD1c^+^ (cDC2) DCs are characterized by BDCA-1 expression [164]. Rather than being evenly distributed across the nasal mucosa, both CD141^+^ and CD1c^+^ DCs are concentrated in particular areas, such as peri-lymphatic, peri-vascular, and sub-epithelial spaces. This strategic positioning enables them to effectively present antigens that enter through the epithelial layer, within the nasal mucosa, and to actively participate in immune surveillance [164]. Hence, the coexistence of these diverse DC subsets in the nasal mucosa implies a collaborative system that ensures effective immune responses and maintains tissue balance.

## 4. Molecular Bridge of Immunity: Unveiling Common CLRs in Macrophages and Dendritic Cells

In the complex field of immunology, where cells play an important role against invasive pathogens, an interesting similarity between two key players stands out as a foundation of immune surveillance. Macrophages and DCs, strategically positioned in the mucosal milieu, share a common array of pattern recognition receptors (PRRs) that enable them to detect the presence of pathogens with remarkable precision. These receptors identify specific molecular patterns, often referred to as pathogen-associated molecular patterns (PAMPs), which are unique to various types of pathogens (bacteria, viruses, fungi, and parasites), as well as patterns associated with cellular damage or stress, known as damage-associated molecular patterns (DAMPs) [165]. PRRs are categorized based on their subcellular localizations: secreted [166], transmembrane [167], and cytosolic receptors [168]. When a PRR detects these patterns, it initiates an immune response to eliminate the invading pathogen. Depending on the PRRs that have been triggered, different immune responses may follow. These responses may include the production of specific cytokines; a direct influence on T-cell polarization, including differentiation into Th1/Th2 cells; T-cell memory formation; and CTL responses. Therefore, this knowledge enables the development of targeted antigen strategies in the nasal mucosa, potentially revolutionizing vaccine development. This approach has the potential to enhance adaptive immune responses and is a way to boost our defenses against a spectrum of diseases, particularly infections transmitted through the respiratory route. Within the category of shared PRRs found in both macrophages and DCs, those that have the most potential as targets for vaccination purposes are mentioned below.

- Toll-like receptors (TLRs)

TLRs are a class of highly conserved transmembrane proteins that act as crucial sentinels of the innate immune system. They include both surface and intracellular receptors. Membrane-bound TLRs (TLR1, TLR2, TLR4, TLR5, TLR6, and TLR10) reside on the cell membrane, while intracellular TLRs (TLR3, TLR7, TLR8, and TLR9) are located in cellular endosomal compartments [169]. Each TLR subtype exhibits specificity for distinct ligands, enabling it to discriminate a wide array of pathogens. For instance, TLR1 recognizes lipopeptides and triacylated lipopeptides; TLR2 identifies lipoteichoic acid (LTA), lipopeptides, glycolipids, and zymosan; TLR3 distinguishes double-stranded RNA; TLR4 recognizes LPS; TLR5 identifies bacterial flagellin; TLR6 distinguishes diacylated lipopeptides and LTA; TLR7/8 recognizes single-stranded RNA; and finally TLR9 identifies unmethylated CpG DNA motifs [170]. TLR10 stands out as a distinctive PRR with the capacity to display anti-inflammatory properties. Studies have revealed that TLR10 can modulate immune responses by mitigating the inflammation typically associated with TLR2 activation. This interplay between TLR10 and TLR2 results in the regulation of pro-inflammatory responses, striking a balance that prevents unwarranted inflammation while maintaining the immune system’s ability to address threats effectively [171]. Although the specific ligands for TLR10 remain less elucidated, there are indications that its ligand recognition patterns are similar to those of TLR1 and TLR2 [172]. Structurally, TLR exhibits an extracellular domain containing leucine-rich repeat (LRR) motifs, responsible for ligand binding, as well as a cytoplasmic toll/interleukin-1 receptor (TIR) domain that can interact with adaptor proteins, initiating downstream signaling cascades. In the case of cell surface TLRs, a transmembrane domain anchors the receptor in the cell membrane [173]. When a ligand binds to the LRR domain of the TLR, it triggers a structural alteration, causing a conformational change. This adjustment brings the intracellular TIR domains into closer proximity, which in turn enables the recruitment of distinct adaptor molecules [174]. The main adaptor molecules associated with TLRs include myeloid differentiation primary response 88 (MyD88) [175], TIR-domain-containing adapter-inducing interferon-β (TRIF) [176], TRIF-related adapter molecule (TRAM) [177], and the TIR-domain-containing adapter protein (TIRAP or MAL) [178]. The activated TLRs then recruit IL-1R-associated kinases (IRAKs), triggering the activation of mitogen-activated protein kinase (MAPK) and nuclear factor-κB (NF-κB) pathways, along with IFN regulatory factors (particularly IRF3 and IRF7) [179,180]. This activation subsequently leads to the expression of cytokines, both pro- and anti-inflammatory, as well as type I interferons, all of which play key roles in shaping and regulating adaptive immunity.

TLRs have emerged as integral components in vaccine design, largely owing to their potent adjuvant capabilities. This adjuvanticity effect not only improves the magnitude of immune responses, but also directs the immune system toward a robust and protective outcome. Interestingly, TLRs have been linked to the efficacy of nasal vaccines, mostly because of their presence in mucosal sites, which facilitates the recognition of PAMPs present in vaccine formulations. Furthermore, certain agonists, like Poly I:C (TLR3 ligand) or resiquimod (TLR7/8 ligand), have been shown to be efficient at eliciting mucosal immune responses, specifically by stimulating antigen-specific T- and B-cell responses [181]. It is worth noting that TLRs’ adjuvant effect extends beyond direct interactions with vaccine components; they often enhance antigen-presenting cell activation and antigen uptake, contributing to the overall immune response to the vaccine. Nevertheless, TLRs are unable to take up antigens directly.

- Retinoic acid-inducible gene I (RIG-I)-like receptors (RLRs)

RLRs are a family of cytosolic pattern recognition receptors in the immune system, primarily responsible for detecting viral RNA and initiating antiviral responses. RLRs are divided into three main members: RIG-I (retinoic acid-inducible gene I), MDA5 (melanoma differentiation-associated gene 5), and LGP2 (laboratory of genetics and physiology 2). Structurally, RLRs share similar domains, including the N-terminal caspase activation and recruitment domain (CARD), a central DExD/H box RNA helicase domain, and a C-terminal regulatory domain [182]. RIG-I senses short viral RNA or 5′ triphosphate RNA, while MDA5 predominantly recognizes long double-stranded RNA [183]. LGP2, although lacking a CARD domain, modulates the activity of RIG-I and MDA5. RIG-I and MDA5 undergo conformational changes that expose their CARD domains when they bind to viral RNA, which makes it easier for them to interact with the mitochondrial antiviral-signaling protein (MAVS) in a homotypic CARD-CARD manner. The engagement between RLRs and MAVS triggers the activation of interferon regulatory kinases, such as TANK-binding kinase 1 (TBK1) and IκB kinase ε (IKKε). These kinases are capable of phosphorylating transcription factors like IRF3 and IRF7. Consequently, phosphorylated IRF3 and IRF7 become activated, and in conjunction with the NF-κB transcription factor, orchestrate the synthesis of type I interferons and prompt the expression of a diverse array of antiviral molecules [184].

Hence, harnessing RLRs as vaccine adjuvants shows potential for inducing robust immune responses and guiding the immune system toward an efficient antiviral state.

- Absent in melanoma 2 (AIM2)-like receptors (ALRs)

AIM2-like receptors belong to a family of cytosolic receptors that detect cytoplasmic double-stranded DNA (dsDNA) [185]. When ALRs sense DNA from pathogens or damaged cells, they can trigger an inflammasome response, leading to the activation of inflammatory caspases, such as caspase-1. This activation results in the release of pro-inflammatory cytokines IL-1β and IL-18 and can potentially culminate in pyroptotic cell death [186]. The structure of AIM2 includes an N-terminal pyrin domain (PYD), which contributes to the formation of inflammasomes through protein–protein interactions, a linker region that allows the proper spatial arrangement of the PYD for inflammasome assembly and a C-terminal HIN domain that contains positively charged amino acids and therefore can bind to dsDNA through electrostatic interactions [187].

Exploiting ALRs’ capacity to trigger the release of these pro-inflammatory cytokines has garnered interest in vaccine design. To take advantage of the adjuvant properties of ALRs, researchers are integrating ALR-activating ligands, such as synthetic DNA analogs or DNA motifs from pathogens, into vaccine formulations.

- Cyclic GMP-AMP synthase (cGAS)/stimulator of interferon genes (STING) pathway

cGAS is a cytosolic DNA sensor that detects DNA from foreign sources like pathogens and endogenous sources like senescent cells. This recognition triggers the generation of cyclic GMP-AMP (cGAMP) through the cyclization of ATP and GTP, which acts as a secondary messenger. Subsequently, cGAMP engages with STING, initiating downstream signaling pathways. This includes the activation of transcription factors IRF3 and NF-κB, resulting in the production of type I interferons [188]. These interferons, in turn, prompt the expression of interferon-stimulated genes that collectively coordinate antiviral defense responses [189].

STING agonists, which activate downstream signaling upon binding to the STING receptor, have demonstrated remarkable potential as adjuvants. By incorporating these agonists into vaccine formulations, researchers aim to trigger their ability to induce robust immune activation and the production of pro-inflammatory cytokines [190,191].

- Nucleotide-binding and oligomerization domain (NOD)-like receptors (NLRs)

NLRs constitute a large family of cytosolic pattern recognition receptors that can detect an array of intracellular signals, ranging from pathogenic components like bacterial, viral, and fungal elements to DAMPs from damaged cells [192,193]. The structure of NLRs comprises three domains: a C-terminal leucine-rich repeat (LRR) domain responsible for ligand recognition, a central NACHT domain (named after NAIP (neuronal apoptosis inhibitor protein), CIITA (class II transactivator), HET-E (heterokaryon incompatibility), and TP1 (telomerase-associated protein) proteins) that exhibits ATPase activity, facilitates oligomerization, and binds nucleotides crucial for NLR activation, and an N-terminal functional domain. The N-terminal domain regulates subsequent signaling events by interacting with adapter molecules containing similar domains [194]. This receptor family can be further categorized into subfamilies based on the N-terminal signaling domain type: PYD domain, a CARD domain, an acidic transactivation domain (AA), or a baculovirus inhibitor of the apoptosis protein repeat domain (BIR). Among these subgroups, the NLRC subfamily, characterized by the presence of CARD domains, and the NLRP subgroup, distinguished by its PYD domains emerge as the two most well-known subfamilies of these proteins, recognized for mediating homotypic domain interactions. Both of these subgroups play a role in the assembly of inflammasomes, which are multi-protein complexes comprising an NLR, the adaptor protein ASC known as the apoptosis-associated speck-like protein, and the effector molecule pro-caspase-1 [195]. It is noteworthy that ASC is composed of two domains: a pyrin domain and a CARD domain. Consequently, NLRC prompts caspase 1 activation via interactions between CARD domains, whereas NLRP activates it through PYD-PYD interactions [196]. Furthermore, following activation, NLRs have been observed to elicit inflammatory reactions, such as NF-κB signaling, which promotes the recruitment and activation of immune cells to the site of infection. They also induce autophagy, contributing to cellular homeostasis, and regulate the expression of MHC class I and II genes [197].

In the context of vaccine design, certain inflammasome-activating ligands have been identified as potential adjuvants due to their ability to induce a hyperactivated state in immune cells, enhancing immune responses without triggering pyroptosis. While inflammasome activation often leads to pyroptosis, a form of programmed cell death mediated by gasdermin D pore formation, certain ligands can hyperactivate cells, allowing them to secrete pro-inflammatory cytokines while maintaining viability. This state of hyperactivation can be leveraged to boost vaccine efficacy. For instance, the synthetic ligand L18-MDP has been shown to hyperactivate immune cells, particularly through cDC2 dendritic cells, promoting a Th1- and Th17-biased immune response. In contrast, stronger inflammasome activators, like ATP, tend to induce caspase-1-dependent pyroptosis, making them less suitable as adjuvants due to the associated cell death [198]. Additionally, Zanoni and colleagues demonstrated that self-encoded oxidized phospholipids induced a hyperactive state in DCs through non-canonical NLRP3 inflammasome activation. These hyperactivated DCs exhibit enhanced survival and IL-1β secretion, making them superior inducers of adaptive cellular immunity, and highlighting their potential in vaccine development and immunotherapy strategies [199].

- Scavenger receptors (SRs)

SRs form an additional class of cell membrane and soluble PRRs. This family includes various distinct groups of receptors that exhibit heterogeneity in terms of structure and/or function. Structurally, SRs encompass a spectrum of classes (A to L), each characterized by distinct extracellular domains responsible for ligand recognition [200]. SRs demonstrate a broad range of ligand-binding properties, engaging with altered self-molecules, DAMPs, and microbial PAMPs (for instance, LTA and LPS). Furthermore, they have the capacity to identify unmodified endogenous biomolecules, such as carbohydrates, phospholipids, cholesterol esters, proteoglycans, proteins, and lipoproteins, which represent essential components of cells and tissues [201,202]. From a broader perspective, scavenger receptors play a fundamental role in recognizing and eliminating undesirable components from the body’s circulation and tissues. This is accomplished through processes like endocytosis, phagocytosis, and macropinocytosis, which collectively contribute to the preservation of internal balance. Moreover, these versatile receptors are now recognized to play additional roles, including the involvement in cellular adhesion and presentation of antigens to MHC class I and II molecules [203,204]. Notably, scavenger receptors can collaborate with other PRRs, such as TLRs, or engage in complex assemblies on various cell types. These collaborations expand their functionalities beyond scavenging alone and extend into areas like cellular signaling. For instance, the receptor CD36 has been documented to not only interact with SRC family kinases, but also to form noteworthy associations with an assortment of transmembrane proteins. These co-receptors include tetraspanins, like CD9 and CD81; TLRs, such as TLR2, TLR4, and TLR6; as well as β1 integrin, β2 integrin, and β5 integrin [205]. Furthermore, SRs possess the capability to operate as lipid transporters, playing a pivotal role in governing the body’s cholesterol balance. Nevertheless, an overabundance of the intake of altered low-density lipoproteins by these PRRs can instigate the creation of foam cells, a defining characteristic of atherosclerosis. Additionally, these receptors have the potential to shape the phenotype of macrophages, acquiring M1 or M2 polarization, depending on the prevailing circumstances [206].

- Complement receptors (CRs)

A large variety of cell surface proteins, including soluble and membrane-bound proteins, is referred to as complement receptors. In mammals, the complement system plays a central role as a crucial effector, combining innate and adaptive immune responses. Complement proteins predominantly exist within the human body in an inactive pro-enzyme state. Upon encountering PAMPs or DAMPs, the complementing PRR domains are activated, establishing an intricate cascade of protease activities [207]. The interaction of these receptors with diverse ligands triggers the induction of three distinct pathways for complement activation: the classical pathway, the lectin pathway, and the alternative pathway. Despite the fact that each pathway’s target detection mechanism is distinct, all three pathways uniformly trigger the activation of C3 and C5 [208]. This, in turn, leads to the generation of various processed complement fragments, like C3a, C3b, C5a, and C5b. Essentially, complement activation causes the recruitment and activation of innate immune cells through C3a and C5a, the direct disruption of the target pathogen by forming the membrane attack complex (MAC) via C5b, and the opsonization of targets through C3b [209]. Therefore, the main functions of CRs include supporting the activity of antibodies and phagocytes through microbial eradication and the clearance of apoptotic cells and debris. They promote antigen opsonization for phagocytosis, enhance immune cell activation, and contribute to the initiation of adaptive immune responses by serving as co-stimulators for B-cell activation and subsequent antibody production [210]. Moreover, CRs actively participate in immune surveillance and tissue repair, the latter being associated with membrane-bound complement regulatory proteins, CD46 and CD55, which prevents excessive complement activation and damage to host cells. Nevertheless, specific bacterial and viral pathogens possess the ability to elude this immune response by interacting with complement inhibitors, evading the effects of complement activation [211].

- C-type lectin-like receptors (CLRs)

CLRs encompass a vast superfamily of over 1000 proteins, each characterized by one or more distinctive C-type lectin-like domains (CTLDs). These proteins have been systematically categorized into 17 subgroups based on their domain organization and evolutionary relationships. CLRs are mostly expressed on myeloid-derived cells, like DCs, macrophages, and neutrophils. Additionally, some CLRs can also be found on non-hematopoietic cells, such as epithelial cells and endothelial cells [212]. Their roles cover a multitude of functions, including cell adhesion, phagocytosis, antigen presentation, platelet activation, cytokine production, the regulation of NK cell activation and cytotoxicity, complement activation, tissue remodeling, immune cell recruitment, and the modulation of immune responses through both activating and inhibitory pathways, among others.

CLRs manifest in two primary forms, soluble and transmembrane CLRs, which are differentiated by their molecular structures. Soluble CLRs constitute a distinctive subset of lectins, characterized by their capacity to dissolve in plasma and their distribution across mucosal surfaces. These soluble receptors have unique compositions and functionalities. Notably, their engagement with microbes can lead to various outcomes, including opsonization, complement activation, initiation of phagocytosis, and inhibition of microbial proliferation. In terms of structure, it is noteworthy that soluble CLRs lack intrinsic signaling motifs. Prominent examples within this category include mannose-binding lectin (MBL) and surfactant proteins [213]. Transmembrane CLRs can be classified into two main types based on their membrane topology: type I and type II transmembrane proteins. Type I receptors share a sequence homology with the mannose receptor and exhibit an outward-facing N-terminus and possess multiple carbohydrate recognition domains (CRDs), which contribute to their broad ligand recognition capacity. In contrast, type II receptors share a homology with the asialoglycoprotein receptor and feature an N-terminus oriented toward the cell’s interior, while their extracellular C-terminus contains merely one CRD [214]. The capacity of CLRs to induce either immune activation or suppression is governed by distinct motifs located in their intracellular domains. Activation of cells occurs through CLRs encompassing immunoreceptor tyrosine-based activation motif (ITAM) domains, which in turn trigger immune responses. Conversely, CLRs containing immunoreceptor tyrosine-based inhibition motif (ITIM) domains typically inhibit cellular activities, supporting regulatory roles [215]. Furthermore, CLRs can also possess a hemi-immunoreceptor tyrosine-based activation motif (hemITAM) or may lack any of these discernible signaling motifs altogether. Upon ligand binding to CLR that contains ITAMs motifs, the presence of receptor-associated protein tyrosine kinases, such as Src family kinases, causes phosphorylation in specific tyrosine residues of the CLR in ITAM sequences. Phosphorylated ITAMs serve as binding sites for signaling molecules, such as Syk and other adaptor proteins, which then initiate a series of downstream signaling cascades. For instance, activated Syk kinase can subsequently phosphorylate and activates CARD9 (Caspase Recruitment Domain-containing Protein 9), which then recruits Bcl10 (B-Cell Lymphoma 10) and MALT1 (Mucosa-Associated Lymphoid Tissue Lymphoma Translocation protein 1) to form the CARD9/Bcl10/MALT1 complex. This complex activates the transcription factor NF-κB, which leads to the secretion of several pro-inflammatory cytokines, contributing to the overall immune response against pathogens [216]. When a CLR bearing an ITIM motif is engaged by its ligand, the receptor becomes phosphorylated on specific tyrosine residues in the ITIM sequence. This phosphorylation leads to the recruitment of protein tyrosine phosphatases, such as SHP-1 and SHP-2, which are enzymes involved in the modulation of signaling pathways, namely by suppressing or attenuating downstream signaling events that would normally lead to immune cell activation [217]. Besides these CLRs’ capacity to independently trigger intracellular signaling cascades upon ligand interaction, they also influence TLR-driven signaling processes, thereby intricately adjusting the magnitude and quality of immune responses. This modulation occurs through diverse mechanisms, including receptor crosstalk, cross-activation, and cooperative signaling [218].

Formerly known for their reliance on calcium ions (Ca^2+^) for recognizing carbohydrates, numerous CLRs exhibit conserved residues in their C-type lectin-like domains. Examples of these include the EPN (Glu-Pro-Asn) and QPD (Gln-Pro-Asp) motifs, which confer binding specificity to mannose and galactose-containing carbohydrates, respectively. However, it is worth noting that CLRs can still recognize sugars and a broader range of ligands, including proteins, lipids, and glycoproteins, even in the absence of these motifs. Furthermore, while many C-type lectin receptors include a CTLD, they do not have any kind of ionic need for ligand recognition. These characteristics led researchers to adopt the phrase “C-type lectin-like domain” as a more inclusive alternative to the initial designation of the “C-type lectin domain” [219].

The activation of APCs by CLRs plays a crucial role in guiding adaptive immune responses. This involves recognition of pathogens through glycan-mediated mechanisms, followed by the internalization of antigens. These antigens are then loaded onto MHC class I and II molecules, consequently steering T-cell polarization through cytokine production and the upregulation of co-stimulatory molecules. Additionally, CLRs contribute to antibody generation and the establishment of immunological memory. These adaptive reactions are essential for preventing reinfection and are integral to ensuring the efficacy of vaccines. Notably, effectively targeting CLRs is of particular significance for developing mucosal vaccines [220]. Hence, it becomes evident that ligands identified by CLRs inherently exhibit adjuvant characteristics. Adjuvants are substances integrated into vaccines to amplify the immune reaction elicited by the antigen. The thoughtful exploitation of CLR-based adjuvants presents substantial promise in molding the future landscape of vaccines, especially concerning improving immune responses against respiratory pathogens.

A diverse array of delivery systems has been intricately designed to effectively target specific immune cells, tailoring delivery methods to pinpoint distinct subsets of APCs and modulate immune responses. These advancements are instrumental in the progressive refinement of nasal vaccine formulations. Having provided an overview of PRRs and their general functions, it becomes evident that CLRs have emerged as particularly promising candidates for targeted vaccine strategies. The strategies for effectively targeting CLRs can be categorized into two primary approaches: firstly, strategies based on the use of natural ligands for these receptors; and secondly, strategies that rely on antibodies specifically designed to interact with these receptors. Immunizations using these natural ligands lack precision, given that numerous CLRs identify comparable carbohydrate structures. Nonetheless, carbohydrate-presenting ligands have the potential to initiate distinct signaling cascades, which could potentially facilitate immune activation. In contrast, antibodies offer a means of precisely targeting CLRs. However, it is worth noting that, while many antibodies can effectively bind to these receptors, they often lack the capacity to trigger robust signaling pathways or might inadvertently induce irregular signaling events [221]. Furthermore, the effectiveness of these ligands may also be significantly hampered due to their potential uptake by other cells through nonspecific Fc receptor (FcR) interactions. Balancing ligand specificity with immune response modulation remains a challenge.

In light of these considerations, our focus now shifts to delve into the specific roles of two distinct receptors within the CLR family. It is of great interest to review the mannose receptor (MR/CD206), a type I transmembrane receptor, as well as the macrophage galactose-type lectin (MGL), which belongs to the type II transmembrane receptor category. By examining these receptors individually, we aim to shed light on their unique contributions to immune responses and pathogen recognition, highlighting what makes them valuable targets for mucosal vaccination purposes.

### 4.1. Mannose Receptors: Insights into Type I Transmembrane CLR Receptors

The MR is found primarily in particular myeloid cell subsets, namely macrophages and DCs (particularly monocyte-derived DCs [222] and cDCs [223]). Additionally, it is important to mention that MR presence extends to certain epithelial and endothelial cell populations, including liver sinusoidal endothelial cells; hepatic, lymphatic, and retinal pigment epithelia; and mucosal epithelial cells [224]. Regarding its expression levels, the MR is subject to variations influenced by multiple factors. These factors include the specific tissue involved, the prevailing physiological conditions, and the immune context. Cytokines, such as IL-4 and IL-13, have been noted to play roles in upregulating MR expression, particularly in scenarios like allergic responses and defense against parasitic infections. Conversely, in the context of infections or tissue injury, transcription factors, such as PPAR-γ (peroxisome proliferator-activated receptor gamma), and cytokines, like TNF-α and IFN-ɤ, can exert differential regulation on MR expression. Consequently, it is essential to recognize that the expression of MR is a highly dynamic process intricately linked to the activation state of the MR-expressing cell [225].

**Figure 2:**
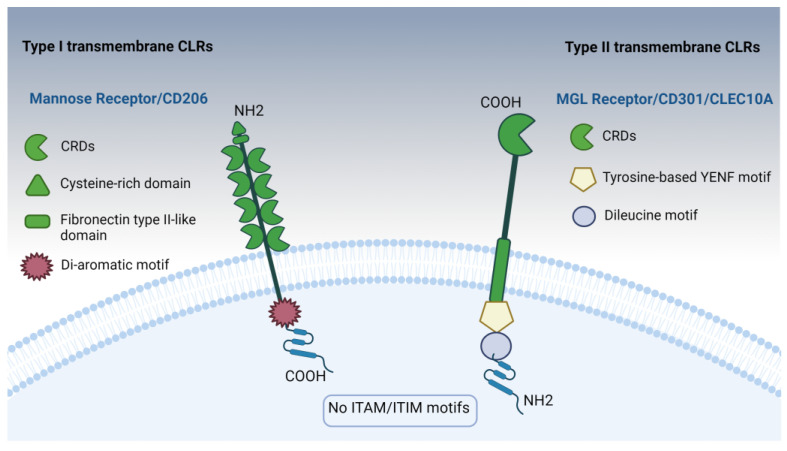
Schematic illustration exhibiting two distinct types of C-type lectin-like receptors (CLRs): type I and type II in an antigen-presenting cell (APC). The mannose receptor, categorized as a type I CLR, reveals a complex structure featuring several components. It comprises an N-terminal cysteine-rich domain, a single fibronectin type II domain, and a sequence of 8 carbohydrate recognition domains (CRDs). Notably, it possesses a di-aromatic motif within its cytoplasmic tail. In contrast, the macrophage galactose-type lectin (MGL) receptor represents a type II CLR with a simpler architecture. It consists of a single CRD, a transmembrane region, and an N-terminal cytoplasmic tail that contains the presence of a dileucine motif and a tyrosine-based YENF motif. Created with BioRender.com.

The MR is a 175 kDa type I transmembrane protein belonging to the CLR family (Figure 2). It finds its place within the extended spectrum of the mannose receptor family, comprising essential receptors, such as Endo180 (CD280) [226], DEC-205 (CD205) [227], and the receptor for phospholipase A2 (PLA2R) [228,229]. The MR′s significance lies in its remarkable ability to recognize distinct carbohydrate moieties, such as mannose, fucose, and N-acetylglucosamine. These carbohydrates are frequently encountered on the surfaces of pathogens and antigens, and their recognition occurs via mechanisms that depend on the presence of calcium ions (Ca^2+^) and through its CRDs [230]. Conversely, the MR also exhibits the ability to detect sulfated acidic glycans, such as chondroitin sulfate [231], glycosylated hormones, and cell surface proteins that can be modified by glycosylation with sulfate groups like CD169 and CD45 by means of its cysteine-rich domain, using a binding mechanism that is not dependent on Ca^2+^ [232]. In terms of its structure, the MR is characterized by three different regions: an extracellular domain featuring eight c-type lectin-like domains responsible for endogenous and exogenous glycoconjugate recognition; a transmembrane domain that secures the MR within the cell membrane, ensuring its stability and proper orientation for ligand binding; and a hydrophilic cytosolic domain, which is involved in the interaction with intracellular signaling molecules that then initiate signaling cascades that regulate cellular responses [233]. The MR′s capacity to bind carbohydrates is predominantly due to specific residues found within its fourth to eighth CRD, with the first three CRDs demonstrating relatively weaker carbohydrate-binding capabilities [234]. Across all these CRDs, there is an ability to interact with mannose-rich oligosaccharides. Nevertheless, it is notable that the fourth CRD exhibits the highest affinity for monosaccharides, such as mannose and fucose [235]. Moreover, the cytoplasmic tail of the MR plays a critical role in regulating its intracellular trafficking, preventing its transport to lysosomes, where it would otherwise be degraded. In particular, MR contains di-aromatic motifs that serve as an endocytic sorting signal. This signal is critical in governing the MR′ clathrin-dependent internalization process and guiding the MR away from the lysosomal degradation pathway, but instead they are recycled constitutively from the endosomal system back to the plasma membrane. This recycling mechanism allows the MR to continue playing its role in pathogen recognition and antigen uptake, by maintaining its presence on the cell surface [236,237]. Interestingly, the extracellular domain of the MR also includes a cysteine-rich domain and a fibronectin type II-like domain that specifically binds collagen (especially types I-IV) [238]. The N-terminal cysteine-rich domain contains multiple cysteine residues, which play a crucial role in forming disulfide bonds. These disulfide bonds contribute significantly to stabilizing the overall structure and integrity of the receptor, particularly in the acidic pH environment encountered during endocytosis, where structural stability becomes decisive for the receptor’s function [239].

In addition to its membrane-bound configuration, the MR can undergo proteolytic cleavage by metalloproteases, resulting in its release into the extracellular milieu as a soluble variant (sMR). This soluble form comprises the entire extracellular domain of MR and is detectable in the supernatant of cells expressing MR, as well as in human serum and extracellular vesicles [240]. Notably, sMR retains the ability to recognize mannosylated and sulfated carbohydrates and plays a decisive role in conveying mannosylated antigens to a specific subpopulation of macrophages found in secondary lymphoid organs. Furthermore, it has been reported that sMR possesses the capacity to directly interact with CD45, a cell surface protein on macrophages. This interaction leads to the inhibition of CD45′s phosphatase activity, subsequently triggering the activation of the Src/Akt signaling pathway and facilitating the nuclear translocation of NF-κB. These events, in turn, contribute to cellular reprogramming adopting an inflammatory phenotype [241].

#### 4.1.1. Ligand Recognition Mechanisms of the MR

The MR′s ligand binding mechanisms are particularly intriguing, as they are intricately linked to both endocytic and phagocytic pathways. In the context of endocytosis, the MR′s journey begins at the cell surface, where it captures glycoproteins and glycolipids decorated with its target carbohydrates, including lysosomal glycosylated enzymes, as part of the cellular process for enzyme recycling. The MR undergoes internalization and is transported into the endosomal system through a process facilitated by clathrin-coated vesicles. This process involves the assembly of clathrin molecules in conjunction with adapter protein complexes, such as AP-2 (Adaptor Protein Complex 2), which recognizes and binds to the sorting signals on the MR′s cytoplasmic tail. As clathrin molecules come together, they form a lattice structure that inherently results in the invagination of the membrane. This assembly results in the formation of vesicles, which are subsequently released into the cell during the final stage of bud formation. These vesicles can then fuse with other endosomal compartments or organelles [242]. Interestingly, upon ligands binding, the MR initiates a transformative shift in its conformation, a process intricately linked to pH variations in its surroundings. Under normal physiological pH conditions, the MR assumes an elongated conformation, which could be advantageous for its ability to capture ligands at the cell’s outer surface. Nonetheless, once the MR-containing ligands is internalized into endosomes, they encounter an increasingly acidic environment. This acidic pH triggers the first step of the conformational change, which consists in a transition of the MR into a more compact conformation. The initial step in this conformational transformation generally occurs at a pH range of 6–7, which aligns with the typical pH of early endosomes [243]. However, considering that some MR ligands may be directed toward late endosomes, characterized by even lower pH levels than early endosomes, it is probable that the second conformational change in MRs occurs within these late endosomes. The endosome’s acidic pH facilitates the release of the MR′s bound ligand. Subsequently, MRs devoid of ligands return to the cell’s membrane, where they can continue their role in ligand capture. Meanwhile, internalized ligands, particularly those linked to antigens, progress to lysosomal compartments and undergo proteolytic degradation, eventually leading to the presentation of processed antigens via MHC II molecules [244]. Furthermore, it was also reported elsewhere that antigens internalized by the MR can be directed toward a specific subset of early endosomes. These particular endosomes exhibit a distinct behavior: they do not promptly fuse with lysosomes and, as a consequence, the antigens internalized via the MR find protection from lysosomal degradation. This protection of antigens is due to the addition of ubiquitin chains to the MR itself and allows an efficient process known as cross-presentation, whereby MR-internalized antigens can be presented via MHC I molecules, leading to the activation of cellular immune responses [245].

Beyond its role in endocytosis, the MR also functions as a phagocytic receptor, promoting the ingestion of large particles, like bacteria (*Mycobacterium tuberculosis* and *Streptococcus pneumoniae*), parasites (*Leishmania*), fungi (*Candida albicans* and *Pneumocystis jirovecii*), viruses (HIV), antigenic particles (>0.5 μm in diameter), or apoptotic cells, and the generation of reactive oxygen species (ROS) [246]. The binding of MR to these particulate ligands triggers the formation of a phagocytic cup, characterized by the invagination of the cell membrane around the particle. This actin-mediated process involves the reorganization of F-actin, a key component of the cell’s cytoskeleton, to facilitate the cup’s closure and the subsequent formation of a phagosome, effectively encapsulating the ingested particle. Once internalized, the phagosome begins to migrate toward the cell’s interior via the endocytic pathway. During this process, F-actin depolymerizes, allowing for dynamic movement. As the phagosome advances, it undergoes maturation by merging with lysosomes, resulting in the formation of a phagolysosome, in which the pathogen or particle faces enzymatic degradation processes, leading to its eventual breakdown and clearance [247].

This unique ability of the MR to bridge endocytic and phagocytic pathways through carbohydrate recognition highlights its significance in immune surveillance and response, presenting the immune system with a versatile tool for identifying and neutralizing a diverse spectrum of pathogens and antigens.

#### 4.1.2. Mannose Receptor Signaling and the Role of Immunopotentiators in Vaccines: A Key to Enhanced Immune Responses

Although MR was one of the first C-type lectin receptors to be studied, its signaling mechanisms have since become a focal point of considerable research. This interest arises primarily from the observation that its cytoplasmic tail does not contain ITAM or ITIM motifs. Consequently, it is suggested that the MR′s signaling functions require the involvement of co-receptors to modulate gene expression and cytokine generation. Numerous reports have provided evidence of the MR′s ability to collaborate with TLR2 or TLR4, resulting in the modulation of cellular immune responses when exposed to pathogenic microorganisms or immunostimulatory agents [248]. Wagener and co-workers demonstrated the collaborative recognition of fungal chitin by MR, NOD2, and TLR9. Together, these receptors orchestrate an anti-inflammatory response characterized by the secretion of IL-10. This response serves to dampen the effects of pro-inflammatory cytokines and redirect the immune response toward a Th2 phenotype [249]. Furthermore, a clear synergy between the MR and Dectin-1 has been revealed when immune cells encounter fungal pathogens, such as *Candida* species. Notably, the presence of *C. albicans* has been linked to the stimulation of IL-17 secretion in an MR-dependent manner. For the elimination of the pathogen, it also contributes the route involving TLR2 and Dectin-1. This pathway is triggered by the recognition of β-glucans on the surface of yeast cells, further amplifying the production of pro-inflammatory cytokines and facilitating an oxidative burst [250]. Nonetheless, the research conducted by Kooij and colleagues highlighted the significant role of MR in modulating protein kinase C (PKC) signaling. This involvement extends to the regulation of processes such as the activation of lymphocyte function-associated antigen 1 (LFA-1), the expression of C-C chemokine receptor 2 (CCR2), and the secretion of cytokines [251]. The MR has also been associated with phosphorylation of its cytoplasmic tyrosine residue when it binds to the cell wall component mannose-capped lipoarabinomannan (found in *M. tuberculosis*). This interaction leads to the recruitment of FcRγ, which, upon phosphorylation, recruits another adaptor molecule known as Grb2. Subsequently, this process activates a complex involving Rac-1, Cdc42, and PAK-1. The activation of PAK-1, Rac-1, and Cdc42 is not only crucial for phagocytosis, but also for processes such as chemotaxis and cell–cell adhesion. These processes are particularly significant for mediating interactions among immune cells within lymphoid organs. Additionally, Rac-1 can stimulate the assembly of the NADPH oxidase complex, resulting in the generation of ROS. Furthermore, MR activation leads to the recruitment of a tyrosine phosphatase called SHP-1 to the phagosome. This recruitment serves to limit the activity of PI3K (phosphoinositide 3-kinase) and inhibits phagosome–lysosome fusion [252]. These findings emphasize the MR′s capacity for direct signaling influence.

In addition to its role in recognizing and processing ligands for cross-presentation by APCs, the cell-surface MR demonstrates the ability to induce antigen-specific tolerance. This aspect becomes particularly relevant in the context of vaccine development. For instance, when the MR on immature monocyte-derived DCs interacts with ligands expressed on apoptotic cells, it triggers the production of anti-inflammatory cytokines, like IL-10 and IL-1Ra, supporting the development of a regulatory phenotype. Furthermore, the MR demonstrated the capacity to elicit T-cell reprogramming and consequent immunological tolerance. This occurs through the interaction of MR on APCs with CD45 on T lymphocytes, where it inhibits CD45’s phosphatase activity. As a result, the upregulation of Bcl-6 (B-cell lymphoma 6) is prevented, while the inhibitory molecule CTLA-4 is upregulated. This CTLA-4 upregulation is responsible for the reduction in T-cell cytotoxic activity, contributing to immune tolerance [253].

To overcome the potential challenge of antigen-specific tolerance in vaccines and to employ MR effectively as a target, a strategic approach involves the incorporation of adjuvants, such as immunopotentiators like TLR agonists, into the vaccine formulation. The MR, primarily functioning as a receptor for efficient antigen internalization, can play a pivotal role in enhancing the uptake of the vaccine components, including antigens. One prominent illustration of this strategy involves the engineering of nanocarriers with mannose ligands, a process achieved through the coating or conjugation of these ligands onto the surfaces of carriers such as nanoparticles or liposomes. This ensures that mannose is promptly recognized by receptors. Importantly, this mannosylation technique not only enhances antigen targeting to MRs, but also extends its reach to other receptors, such as DC-SIGN (CD209), Langerin (CD207), and DEC-205 (CD205), consequently amplifying the immune response. However, it is worth noting that these receptors exhibit a greater affinity for more intricate mannose ligands as opposed to ligands carrying one or two mannose moieties at their terminals. As an example, the hepatitis B surface protein antigen (HBsAg) was encapsulated into nanocarriers composed of a mannose-decorated PLGA polymer. Following a single immunization, the vaccine utilizing mannose-modified nanoparticles demonstrated notably higher levels of antigen-specific serum IgG titers and a robust cellular immune response, characterized by increased IFN-γ secretion. In contrast, both HBsAg alone and the nanocarrier system without the mannose component exhibited significantly lower immune responses [254]. Shi and colleagues also reported the development of a new delivery strategy based on chitosan nanoparticles containing mannose residues on their surfaces. This approach was designed for targeted delivery to DCs and involved loading the nanoparticles with tumor cell lysates obtained from B16 melanoma cells. Following subcutaneous administration, this formulation was efficiently taken up by DCs and led to a significant increase in the levels of IFN-γ and IL-4 in the serum. Notably, in mice treated with this cancer vaccine, tumor growth was markedly delayed due to the robust response of cytotoxic T cells, highlighting the potential utility of this approach in the field of cancer immunotherapy [255]. Pei and co-workers developed a mannose-functionalized particle by conjugating ovalbumin with mannose and complexing it with polyethylenimine (PEI). This design significantly increased antigen presence in target cells, facilitated endosomal/lysosomal escape, and promoted the production of pro-inflammatory cytokines and DC maturation in vitro. Additionally, when B3Z T-cell hybridoma was incubated with DCs treated with these particles, MHC-I antigen presentation was enhanced [256]. At the opposite end of the spectrum, Su and associates created a novel delivery system that combines the encapsulating properties of liposomes with the practical benefits of mannose-functionalized polymers. This system aimed to enhance the cytosolic delivery of streptomycin to alveolar macrophages. The outcomes of their research showed an increase in antibiotic release, resulting in enhanced antibacterial activity in vitro, when evaluated against the standard liposome formulation. This approach demonstrates potential as a future treatment for pulmonary infections affecting the macrophages [257]. However, to further shape the immune response in a desirable direction, the inclusion of immunopotentiators, like TLR agonists, becomes crucial. These adjuvants stimulate specific immune signaling pathways that can lead to an immune response shift. TLR agonists are known to promote a Th1 and cytotoxic T lymphocyte responses, which is characterized by enhanced cellular immunity, and the production of pro-inflammatory cytokines. This immune response profile is particularly effective for preventing the entrance of pathogens and providing long-lasting protection. Moreover, in the case of employing a nanocarrier decorated with mannose ligands, it facilitates the integration of various immune-stimulating elements within the same carrier, thereby creating a versatile platform for enhancing vaccine efficacy. In a meticulous study conducted by Wilson and associates, a synthetic polymeric adjuvant was created by combining monomers targeting the MR in APCs with a TLR7 agonist. The formulation was administered intradermally and resulted in a substantial increase in the humoral immune response and antigen-specific CD8^+^ T-cell expansion with enhanced cytotoxic activity. The enhanced effect was attributed to the high antigen localization in the draining lymph nodes, which avoided systemic inflammation when compared to the antigen alone or formulations without mannose targeting. Furthermore, the vaccine platform demonstrated its efficacy by reducing malaria parasite levels in human hepatocytes [258]. Zhu and a team of researchers developed a hybrid delivery system that combined synthetic block copolymers and lipids to form vesicles. These vesicles were designed to co-deliver a model antigen, with a TLR7/8 agonist incorporated into the hydrophobic part of the vesicle membrane and a TLR4 agonist located in the lipid layer. This design aimed to synergistically enhance immune responses and improve vaccine efficacy. The polymers were modified with mannose, which facilitated efficient internalization by DCs. In contrast, counterparts lacking the mannose moiety exhibited less activated DCs, with a lower capacity to migrate to lymph nodes. After three intramuscular administrations of the vaccine in mice, a strong cellular response with a Th1-biased immune response was observed. Furthermore, when used prophylactically, the vaccine notably postponed tumor development, showcasing enduring immune responses [259].

The reviewed studies indicate that mannosylated nanocarriers can improve antigen uptake and stimulate both antibody-mediated and cell-mediated immune responses. This finding, coupled with the high expression of the MR in mucosal tissues (such as nasal, pulmonary, and enteric mucosa), underscores the potential relevance of this receptor for the development of mucosal vaccine approaches. Although mannose-mediated targeting alone may not resolve all the obstacles associated with mucosal vaccination, such as suboptimal mucus penetration and antigen retention, functionalizing polymers with mannose can augment their internalization efficiency while concomitantly enhancing the inherent characteristics of the base polymer. For example, Cui and colleagues engineered mannose-decorated chitosan microspheres loaded with an antigen against *Pseudomonas aeruginosa*, a microbe that commonly infects mucosal tissues. When they administered this mannosylated formulation and a non-mannosylated control intranasally to mice, the mannosylated version elicited a markedly stronger mucosal immune response, as evidenced by elevated secretory IgA levels in nasal secretions and other distant mucosal sites. Furthermore, the mannosylated formulation stimulated a more robust Th1-skewed cellular immune response, which is advantageous for therapeutic vaccines [260]. This investigation illustrates that mannose conjugation can improve the performance of chitosan-based platforms for mucosal vaccine administration. Chitosan’s intrinsic mucoadhesive characteristics appear to enhance retention within mucosal environments, enabling more effective internalization via MRs on APCs relative to unmodified chitosan. In another study, Zhuang et al. conducted a study investigating an mRNA vaccine against H1N1 influenza, utilizing cationic lipid nanoparticles and mannose-conjugated cationic lipid nanoparticles. After administering the vaccine formulations intranasally to C57BL/6 mice, the researchers assessed and compared the resulting immune responses. The mice were subsequently challenged with intranasal exposure to the influenza virus. The findings indicate that the mannose-functionalized vaccine provided enhanced protection, as evidenced by the prevention of weight loss and mortality, a significant reduction in lung viral titers, and decreased infiltration of inflammatory cells in the lung tissue, thereby demonstrating its strong mucosal-level protection [261]. While mannosylated vaccines have proven effective as nasal vaccines, they also show potential for oral administration, making them a versatile strategy for mucosal immunization across diverse administration routes. For instance, Wang and co-workers examined the use of mannose-PEG-cholesterol-decorated liposomes as an oral delivery system for a Hepatitis B vaccine administered to mice. This formulation stimulated a robust, balanced Th1/Th2 immune response, with high titers of IgG in the serum and IgA in vaginal, intestinal, and salivary secretions [262]. These findings suggest that the mannosylated liposomal vaccine can serve as a potent prophylactic against HBV, which is transmitted through mucosal surfaces, and also as an effective therapeutic capable of clearing chronic HBV infections due to its induction of cell-mediated immunity.

### 4.2. Macrophage Galactose-Type Lectin (MGL) Receptor (CD301/CLEC10A): Insights into Type II Transmembrane CLR Receptors

MGL, also referred to as CLEC10A or CD301, exhibits a restricted expression pattern primarily limited to myeloid immune cells, specifically macrophages and DCs, within tissues like the skin, lymph nodes, and mucosal regions. The receptor is present on a specific subset of DCs known as CD1c^+^ DCs, and activated M2 macrophages, although the receptor’s expression is often subject to regulation based on the maturation state of APCs [263,264].

In mice, researchers have identified two homologs analogous to the human MGL receptor, referred to as MGL1/CD301a and MGL2/CD301b. MGL1 exhibits a distinct preference for Lewis X and Lewis A structures, whereas MGL2, similar to its human counterpart, displays recognition capabilities for N-acetylgalactosamine (GalNAc) and galactose (Gal) residues. This divergence in ligand binding specificity can be attributed to the presence of secondary binding sites within the CRDs of MGL1 and MGL2 [265]. Interestingly, MGL1 and MGL2 exhibit distinct tissue distribution patterns. MGL1 demonstrates a broader expression profile, being found on macrophages, cDCs, and pDCs. In contrast, MGL2 is primarily localized in cDCs, with a more limited distribution across other immune cell types [266]. Therefore, although human MGL and murine MGL receptors share significant structural and functional similarities, considerable differences exist, particularly in glycan-binding specificities and tissue distribution. These differences are more pronounced when comparing human MGL with murine MGL1, which has distinct preferences for glycan ligands. However, a recent study by Gabba and colleagues also highlighted subtle variations in ligand interactions between human MGL and murine MGL2, emphasizing the importance of species-specific receptor–ligand dynamics [267]. These variances emphasize the importance of studying both human and mouse MGLs to gain a comprehensive understanding of lectin-mediated immune responses and the potential for translational research in immunology and vaccine development.

MGL receptors possesses robust endocytic capabilities, facilitating the ligands rapid internalization from the cell surface into intracellular compartments. This intrinsic feature makes it an excellent candidate for the targeted uptake of antigens and the subsequent transport of these to endosomes, where they can undergo processing and eventual presentation by MHC molecules.

From a structural perspective, MGL is categorized as a type II transmembrane C-type lectin receptor (Figure 2). This designation arises from the presence of a carbohydrate recognition domain located in its extracellular domain. This CRD is characterized by a distinctive QPD motif, which assumes responsibility for the receptor’s selective recognition of glycan structures bearing GalNAc and Gal residues. These glycan structures, present on glycosphingolipids and glycoproteins, are typically found on the surfaces of pathogens, allergens, and host cells, including self-antigens and cancer cells, often characterized as altered self-antigens [268]. Particularly noteworthy is the association of tumor-derived MUC1 and MUC2 glycoproteins with the presence of Tn antigens on their surface. The Tn antigen is a simple carbohydrate structure, comprising a GalNAc residue linked to a serine or threonine amino acid within protein chains, and exhibits a strong binding affinity for MGL. In cancer cells, abnormal glycosylation processes can lead to the exposure of Tn antigens on the surface of mucins. This altered glycosylation is a characteristic feature of many tumors and can contribute to immune recognition, making it a significant aspect of cancer biology [269]. By binding to these structures, MGL facilitates their uptake by DCs and macrophages, promoting phagocytosis, antigen presentation to T cells, and the modulation of immune cell activation. This process is instrumental in initiating adaptive immune responses and generating specific immune memory. However, in some cases, ligand binding to MGL might be also associated with tumor evasion of the immune system through the suppression of immune cell activation and/or the promotion of immune tolerance, which means that the immune system may not recognize the cancer cells as effectively as it should. For instance, Tn antigens and their binding to MGL have been associated with poor prognosis in breast cancer [270] and other adenocarcinomas [271], indicating a significant role for MGL^+^ antigen-presenting cells in tumor progression. This process is not however uniform across all cancer types, depending on several factors such as the tumor microenvironment [272,273,274]. Napoletano et al. conducted an insightful investigation into the interactions between the ovarian cancer-specific O-glycoproteome and the MGL receptor. Their findings showcase the diverse ligands’ affinities for this receptor and elucidate the impact of the tumor microenvironment on immune regulation and cancer progression [275].

In addition to the extracellular CRD domain in MGL, a neck domain is also present. This neck domain plays a pivotal role in maintaining the structural stability of the receptor and acts as a connection between the CRD and the transmembrane domain. In this neck region, a trimeric structure is formed, primarily stabilized by the helical coiled-coil domain. Interestingly, the CRDs located within this region operate as autonomous domains, contributing to the receptor’s ability to recognize various glycan structures [276]. Equally important is the intracellular MGL domain, which harbors two significant signaling motifs. These intracellular motifs play essential roles in transmitting signals and coordinating cellular responses upon ligand binding. The two main motifs are a dileucine motif and a tyrosine-based YENF motif, both of which are common endocytic signals found in many transmembrane receptors [277,278]. These motifs serve as recognition signals for intracellular adaptor proteins, such as clathrin-associated sorting proteins and other sorting machinery. This interaction ultimately determines whether the receptor is recycled to the cell membrane for additional ligand binding or directed toward lysosomal degradation [279]. This mechanism ensures the proper regulation of the MGL receptor’s function and the fate of its ligands in the cell [280].

#### 4.2.1. Understanding MGL Signaling Networks: Shaping the Immune Reaction

To gain an insight into MGL signaling networks and understand their profound influence on immune responses, it is essential to explore how this receptor orchestrates cellular communication and coordinates immune activities. MGL’s intricate signaling pathways are instrumental in modulating immune cell activation, antigen presentation, and immune tolerance mechanisms. By examining these signaling networks in detail, we can obtain a better understanding of the critical function that MGL plays in shaping immune responses, ranging from the recognition of glycan structures to the fine-tuning of adaptive immunity.

There are several reports that emphasize MGL’s key role in antigen presentation to T cells as a fundamental step in initiating adaptive immune responses. For instance, in an elegant study conducted by Napoletano and colleagues, monocyte-derived DCs (artificially generated in vitro) were employed to investigate the interaction of MGL with two distinct ligands: an anti-MGL antibody and an MUC1-Tn peptide. Their investigation revealed that both of these ligands effectively promoted the upregulation of maturation markers, including HLA-DR, CD83, CD40, and the migration marker CCR7. Remarkably, the interaction of MGL with these ligands resulted in the activation of antigen-specific CD8^+^ T cells, as evidenced by the generation of IFN-γ. Additionally, the study observed an enhanced production of IL-12 relative to IL-10 within this DC subset, suggesting their potential to bias polarization toward a Th1 population. Notably, the MUC1 peptide exhibited a more robust activation of intracellular signaling pathways, such as the phosphorylation of ERK1/2 (Extracellular Signal-Regulated Kinases 1 and 2), and the activation of the NF-κB pathway, showing great promise as an adjuvant for the design of innovative anticancer vaccines [281]. Interestingly, in a related study, Heger et al. used ex vivo primary CD1c^+^ DCs isolated from healthy donor PBMCs to compare the immunogenicity of glycosylated versus non-glycosylated MUC-1 epitopes. Their findings demonstrated less pronounced differences between the ligands compared to the previous study. However, they observed that the glycosylated form, when combined with TLR stimulation, elicited the increased secretion of the cytokines TNF-α, IL-10, and IL-8, an effect not observed with the non-glycosylated version [263]. Also, a study in the Netherlands revealed that MGL possesses the capability to target ligands for degradation and their subsequent presentation on MHC class II molecules, ultimately leading to the activation of responding T cells. Importantly, the internalization process relies entirely on the tyrosine-based YENF motif found within the MGL cytoplasmic tail. In contrast, the dileucine motif, also present in the MGL cytoplasmic tail, is believed to be involved in the regulation of subsequent intracellular cargo trafficking [282]. Moreover, research conducted by Singh and co-workers revealed that, when a model antigen, ovoalbumin, was modified with tumor-associated glycans containing GalNAc, it exhibited efficient internalization by DCs via an interaction with MGL2. Subsequently, these DCs presented the antigen, both through MHC class II and class I molecules, to activate CD4^+^ T cells and CD8^+^ T cells, respectively. This antigen presentation led to the differentiation of Th1 cells that produced IFN-γ. Remarkably, this effector T-cell activation was found to be independent of TLR signaling and was solely mediated by glycan interactions [283].

In addition to its well-documented role in antigen presentation, MGL also exerts significant immunomodulatory effects, in different immune contexts. It has been revealed that an engagement with MGL triggers Syk activation in an SFK (Src Family Kinase)-dependent manner. Notably, MGL lacks ITAM or hemi-ITAM motifs in its cytoplasmic tail. However, the presence of positively charged arginine and lysine residues suggests a possible interaction with ITAM-containing transmembrane adaptor proteins, leading to Syk recruitment, similar to observations for several other CLRs that lack internal signaling motifs. As a consequence, MGL specifically triggers the activation of MAPK family members, leading to the subsequent activation of downstream targets, such as p90RSK and CREB. This signaling cascade ultimately leads to the increased expression of IL-10. This, in turn, contributes to the induction of antigen-specific regulatory T-cell responses [284]. It is important to note that the ligand used to interact with MGL, in this context, was an antibody, and that could be one of the reasons for why, when Syk was phosphorylated and activated, it did not lead to NF-κB activation. Interestingly, in a different study, Vliet and co-workers showed that the binding of carbohydrates to MGL not only increased the secretion of IL-10, but also amplified the production of TNF-α. This effect was attributed to the engagement of TLR-mediated signals and the involvement of the NF-κB signaling pathway [285]. Furthermore, one cannot forget that, while IL-10 is known for its anti-inflammatory effects, it also plays a crucial role as a growth factor for CD8^+^ T-cell immunity. Additionally, it contributes to humoral immune responses by influencing the proliferation, differentiation, and survival of human B cells. IL-10 achieves this by upregulating the expression of the anti-apoptotic protein Bcl-2 [286]. Another compelling example of how MGL plays a pivotal role in immune regulation is its capacity to attenuate T-cell immune responses, thereby preventing tissue damage in the context of chronic inflammatory conditions. This mechanism involves the interaction between MGL and the Tn antigen present on CD45 molecules found on activated effector T cells. Consequently, this interaction leads to a reduction in the phosphatase activity of CD45, resulting in several immune-regulatory outcomes [287,288]. Specifically, this process contributes to the downregulation of T-cell proliferation, a decrease in the production of inflammatory cytokines, and an increase in T-cell apoptosis [273,274,275,276,277,278,279,280,281,282,283,284,285,286,287,288,289]. These combined effects represent an essential facet of immune regulation mediated by MGL, highlighting its role in modulating T-cell homeostasis and restraining potentially harmful T-cell activation [290].

Certain ligands that interact with MGL possess a unique ability to evade the human immune system, presenting a complex challenge in the context of immunology. For example, a study by Sorge et al. highlighted the remarkable ability of *Campylobacter jejuni* bacteria to produce intricate carbohydrate structures, including distinct lipooligosaccharide glycoforms and N-linked glycoproteins. These components of the bacteria exploit the endocytic pathway mediated by MGL, effectively evading immune surveillance. Notably, when these ligands bind to MGL, they induce immunosuppressive effects, as evidenced by the modulation of pro-inflammatory cytokines. The authors demonstrated that the production of IL-6 in DCs was significantly reduced when exposed to *C. jejuni*-containing MGL ligands, in contrast to *C. jejuni* strains lacking such ligands [291]. Furthermore, there is evidence to suggest that MGL can serve as a potential entry receptor for various viruses. MGL has been observed to interact with specific glycan structures present on viruses such as the terminal galactose residues of the influenza virus, the densely glycosylated spike protein of SARS-CoV-2, and the viral envelope glycoprotein of the Ebola virus. This MGL-mediated interaction improves the infectivity of these viruses by facilitating their attachment to local cellular receptors, thereby promoting viral entry and infection [292]. All of these mechanisms illustrate how pathogens can exploit MGL to manipulate the host immune response for their benefit. Investigating these elaborate processes is of utmost importance for developing strategies to overcome immune evasion and enhance immune responses against complex targets.

#### 4.2.2. Unlocking MGL’s Potential: Lactobionic Acid as a Promising Ligand for Vaccination

The multifaceted MGL receptor demonstrates its versatility not only in binding various glycan structures, but also in initiating distinct signaling pathways based on the nature of its ligands. The characteristics of ligands, including affinity, avidity, size, density, and overall structure, play a pivotal role in dictating the signaling cascades triggered by MGL engagement. These ligand-dependent signaling phenomena, including receptor clustering and the formation of endocytic synapses, are typical of MGL, yet they are also common characteristics observed across other CLRs. For instance, the formation of a phagocytic synapse, often involving the exclusion of membrane phosphatases and facilitating Syk association and signaling, is not exclusive to MGL, but is also seen in other CLRs [293]. However, depending on the ligand, as seen in the detection of soluble ligands, the inhibitory effects of membrane tyrosine phosphatases may persist with the receptors, resulting in the attenuation of CLR signaling. One of the most well-known examples illustrating this concept can be found in the case of Dectin-1 (also a transmembrane type II CLR) that, when interacting with particulate β-glucans, initiates the creation of a phagocytic synapse, enhancing the efficiency of fungal engulfment and activating a robust immune response by membrane phosphatases exclusion. The same does not happen when dectin-1 interacts with soluble β-glucans [294].

Lactobionic acid, a chemical compound composed of gluconic acid and galactose residues, is an emerging ligand for functional modifications aimed at targeting MGL. However, it is essential to note that the asialoglycoprotein receptor (ASGPR), abundantly expressed on hepatocytes, is also capable of recognizing and internalizing Gal and GalNAc residues. Consequently, the choice of the administration route becomes a critical consideration. For instance, when using a galactosylated drug delivery system via injection, rapid liver absorption is likely due to the high density of ASGPR. In contrast, mucosal vaccine formulations, whether administered nasally or orally, offer a strategy to enhance MGL targeting without significant liver uptake [295]. These routes expose vaccines to the NALT and GALT, both rich in APCs like DCs and macrophages. This proximity to APCs increases the likelihood of efficient antigen presentation and immune responses while minimizing direct ASGPR exposure. Nasal administration, by favoring the interaction with APCs over ASGPR, promotes specific MGL targeting, enhancing desired immune responses while avoiding off-target effects associated with hepatic uptake. While the choice of administration route is undeniably critical, it is important to note that specific contexts, such as the presence of tumors, can introduce unique considerations. For example, in the microenvironment of many solid tumors, there is the presence of tumor-associated macrophages that exhibit elevated levels of MGL expression. Under these circumstances, even with parenteral administration, cancer vaccines can effectively target MGL-expressing immune cells. This illustrates that the expression patterns of lectin receptors, like MGL, can vary across different tissues and conditions, influencing the choice of administration route for optimal vaccine delivery [296].

The application of lactobionic acid-modified delivery systems for targeted drug delivery has gained increasing attention in recent years. Nevertheless, the available literature in this field is somewhat limited, primarily focusing on therapeutic agents rather than vaccines. However, the following examples effectively demonstrate how lactobionic acid-based targeting strategies can guide both therapeutic agents and antigens to the MGL receptor, showcasing the versatility of this approach. While some cases emphasize therapeutic agents, they offer valuable insights into the potential applications of lactobionic acid in vaccine delivery systems. For instance, Zhang and colleagues engineered chitosan nanoparticles decorated with lactobionic acid and loaded with a small interfering RNA (siRNA). These nanoparticles were designed to specifically target activated macrophages, commonly found at inflammatory sites within the colon, by using the MGL receptor for internalization. Within these specialized immune cells, the siRNA cargo was employed to silence the functional protein Map4k4, a key regulator in the TNF-α signaling pathway. Following the oral administration of these nanoparticles, they successfully suppressed TNF-α production within activated macrophages in a mouse model of ulcerative colitis. Cytokines, such as TNF-α, are strongly implicated in the pathogenic processes underlying inflammatory bowel diseases [297]. Similarly, two other studies adopted a comparable methodology to target the same disease, employing lactobionic acid as the ligand for targeting the MGL receptor. Huang and co-workers designed nanoparticles composed of polylactic-co-glycolic acid (PLGA) coated with chitosan that had been previously modified with lactobionic acid. These nanoparticles were further loaded with TNF-α siRNA. When administered orally to mice with colitis, these nanoparticles exhibited efficient uptake by intestinal macrophages, displaying a pronounced affinity for colon tissue over other cell types. Additionally, their findings indicated a therapeutic effect, as evidenced by reduced levels of TNF-α observed in the colonic tissue of the treated mice [298]. Moreover, Xiao and colleagues introduced an innovative approach for addressing intestinal inflammation. They developed a combination therapy using galactosylated polymeric nanoparticles carrying TNF-α siRNA and interleukin-22 (IL-22) incorporated within a chitosan and alginate-based hydrogel. Following the oral administration of these formulations to mice, the study revealed a synergistic therapeutic impact arising from this dual strategy. Notably, there was a substantial increase in the targeted delivery of TNF-α siRNA to macrophages, especially when compared to non-functionalized nanoparticles. This outcome underscored the effective targeting of MGL and the promising potential of the treatment approach [299]. Nevertheless, it is important to note that these reports did not directly demonstrate MGL receptor interaction, but relied on indirect methods. For example, competition assays using free galactose or lactobionic acid in cell lines known to express MGL showed reduced particle uptake when competitors were added, suggesting MGL involvement in internalization. Additionally, studies comparing galactosylated (lactobionic acid-decorated) particles with non-galactosylated ones observed a significantly enhanced macrophage uptake of the former, indicating receptor-mediated internalization, inferred to involve MGL. These findings align with results from Zuo et al., who used galactosylated low-molecular-weight chitosan (which shares the same galactose moiety as lactobionic acid) to form nanocomplexes, and demonstrated increased transfection efficiency upon LPS stimulation of macrophages, linked to increased MGL expression [300]. This further supports the role of MGL in particle uptake. Going forward, the direct assessment of MGL receptor expression will be crucial to better understand the mechanism of lactobionic acid ligation and its role in targeting. In the field of vaccine research, analogous techniques have surfaced, employing MGL targeting to strengthen immune responses and facilitate the development of more potent vaccines. A study conducted by Wu and colleagues demonstrated an original approach involving the chemical conjugation of lactobionic acid to low-density lipoprotein (LDL) nanoparticles containing an encapsulated model antigen. This innovative system exhibited a remarkable capacity for selective delivery to Kupffer cells, a specialized subset of macrophages predominantly residing in the liver. Importantly, this approach not only facilitated the targeted delivery of antigens, but also significantly enhanced antigen presentation through MHC class I molecules, ultimately promoting the activation of antigen-specific T cells. However, it is noteworthy to mention that the precise mechanism responsible for this macrophage targeting was attributed to the interaction with galactose particle receptors. Unfortunately, the study did not specify whether this interaction primarily involved the MGL, the ASGPR, or a potential combination of both receptors [301]. In another elegant investigation, Huang and collaborators developed a sophisticated nanocomplex with the specific aim of targeting tumor-associated macrophages, which closely resemble alternatively activated M2 macrophages. These cells are characterized by their increased expression of the MGL receptor. This innovative nanocomplex consisted of cationic dextran conjugated with lactobionic acid and combined with a TLR9 agonist and anti-IL10 receptor oligonucleotides. The formulation successfully reprogrammed the macrophages toward an M1 phenotype, thereby enhancing cytotoxic T-cell function, and ultimately achieving the efficient suppression of tumor cells. Importantly, this targeting strategy proved highly localized, without triggering an undesirable systemic response [302].

The reviewed studies mainly focused on using lactobionic acid-based systems for oral immunization. To our knowledge, there are currently no reports on the use of lactobionic acid-functionalized nanocarriers specifically for nasal vaccination. However, some studies have explored delivery systems conjugated with galactose, the active component of lactobionic acid responsible for binding to MGL receptors, for nasal administration. These findings suggest that, while lactobionic acid itself has not been widely applied in nasal vaccines, its galactose component has shown potential for targeting MGL receptors in mucosal tissues, indicating it as a promising approach for mucosal vaccine development. For example, Jiang et al. developed a mucosal vaccine employing galactosylated liposomes to target DCs for cancer immunotherapy. This vaccine was administered intranasally and demonstrated a superior performance compared to unmodified liposomes, eliciting robust ovalbumin-specific IgG antibody production in the bloodstream. The vaccine also elicited strong Th1 and Th2 cellular and humoral immune responses, resulting in full protection against tumor growth in C57BL/6 mice. Furthermore, the study demonstrated an enhanced uptake of galactosylated liposomes by DCs in NALT, mediated by the MGL interaction, highlighting the potential of galactose conjugation for mucosal vaccine targeting [303]. In a related study, Wang et al. designed galactose-functionalized liposomes as a targeted delivery platform for macrophages in mucosal tissues. The internalization of these liposomes was evaluated in RAW 264.7 immune cells, where they exhibited a significantly higher uptake compared to unmodified liposomes, likely due to MGL interaction. Following intranasal administration in mice, the galactosylated liposomes elicited significantly elevated levels of ovalbumin-specific secretory IgA in both lung and nasal washes, in addition to an increased production of pro-inflammatory cytokines, highlighting their enhanced potential for mucosal immune stimulation [304].

Therefore, these innovative approaches pave the way for the development of next-generation vaccine delivery systems that can precisely target immune cells and enhance the effectiveness of vaccines against various diseases. The journey to unlock MGL’s potential as a crucial player in vaccination is unfolding as we continue to understand the complexities of lectin receptor-based targeting, promising to shape the future of immunization and boost our defenses against a variety of pathogens.

## 5. Conclusions and Future Perspectives

In conclusion, mucosal vaccines, particularly those administered via the nasal route, show great promise in revolutionizing immunization strategies. The challenges encountered in the development of mucosal vaccines are indeed a worthwhile pursuit, given that the potential gains in terms of immunological advantages and logistical convenience significantly surpass these hurdles. Compared with systemic immunization, mucosal vaccines have the advantage of eliciting robust mucosal and systemic immune responses simultaneously, they are non-invasive, and they offer the opportunity to simplify vaccination programs by reducing the need for healthcare professionals to administer them. The nasal mucosa, with its unique immune-related environment, offers a strategic entry point for vaccine delivery. The utilization of CLRs, such as the mannose receptor and MGL receptor, has emerged as a cutting-edge approach to enhance antigen delivery and uptake by APCs. This targeted delivery not only improves the efficiency of immunization, but also provides a means to tailor immune responses for specific pathogens.

Moving forward, the future of mucosal vaccines lies in their ability to maximize the potential of CLRs while ensuring the appropriateness of immune responses. Combining CLR-targeted antigen delivery with immunomodulators like TLR agonists has tremendous potential. TLR agonists can act as immuno-enhancers, promoting the maturation of APCs and guiding immune responses toward a balanced Th1/Th2 profile. This balanced response is essential for effective immunity against various pathogens, as it ensures both cellular and humoral components of the immune system are engaged.

Furthermore, ongoing research into the development of glycoconjugate vaccines and the application of glycan-based ligands, such as lactobionic acid and mannose, offer exciting avenues to optimize mucosal vaccine formulations. These strategies can enhance the targeting of APCs and boost the overall efficacy of nasal immunizations.

It is important to note that, while the focus is on enhancing antigen delivery to APCs, the formulation should not be directed exclusively to one particular type of APC. The immune system employs a diverse array of APCs, including DCs, macrophages, and B cells, each with distinct roles in initiating and regulating immune responses. Therefore, an ideal mucosal vaccine formulation should be designed to engage multiple types of APCs. Glycoconjugate vaccines, which combine antigens with specific glycan motifs recognized by various APC receptors, can achieve this broad-spectrum targeting. For instance, the creation of a hybrid particle that incorporates ligands, such as mannose and lactobionic acid, within the same delivery system presents an innovative approach. This hybrid particle can effectively engage both mannose receptors and MGL receptors, which are expressed on macrophages and dendritic cells, allowing a simultaneous interaction with these crucial APCs. Despite the potential of this therapy, a comprehensive understanding of how these ligands can effectively trigger an immune response and the specific immune signaling pathways they activate in APCs remains an essential area of investigation. A deeper comprehension of the intricate mechanisms elicited by these ligands will contribute to the design of more precise and effective mucosal vaccine formulations.

## Figures and Tables

**Table 1:** Approved vaccines for human use targeting mucosal surfaces.

Vaccine Name	Disease Targeted	Administration Route	Vaccine Components	Major Features: Mode of Action	References
Dukoral	Cholera	Oral	Killed whole-cell monovalent vaccine composed of formalin or heat inactivated *V. cholerae* O1 strains with the recombinant cholera toxin B subunit (CTB)	Induction of O1 LPS and CTB-specific IgG and IgA antibodies in serum; mucosal vaccine-specific IgG and IgA antibodies, particularly in the nasal cavity; increased number of memory B lymphocytes in circulation that express markers for homing to the small intestine, colon, and airways; poor cell-mediated immune responses	[10,11,12]
ShanChol	Cholera	Oral	Killed whole-cell bivalent vaccine: formalin and heat inactivated *V. cholerae* O1 and O139 strains	Production of gut O1 and O139 LPS-specific IgA and IgM antibodies; systemic IgG antibodies were also produced in the bloodstream, although to a smaller extent than in the intestinal environment	[13,14]
Euvichol	Cholera	Oral	Bivalent inactivated vaccine: formalin and heat inactivated *V. cholerae* O1 (two Inaba and two Ogawa serotypes) and O139 strains	Robust immune response characterized by high levels of vibriocidal antibodies, similar to those induced by the Shanchol vaccine; reduced production costs	[15,16]
Vaxchora	Cholera	Oral	Live attenuated monovalent vaccine: *V. cholerae* O1 strain, serotype Inaba, where the toxigenic A1 subunit of the cholera toxin was removed, leaving only the non-toxic but immunogenic B subunit; contains cryoprotectants (sucrose and hydrolyzed casein), antioxidant (ascorbic acid), and a stabilizer (sodium chloride)	The live attenuated cholera bacteria undergo replication within the gastrointestinal tract leading to the production of anti-CTB and anti-LPS specific IgG antibodies in the serum; enhancement of anti-O1 LPS IgA and IgG memory B cells; presence of anti-LPS IgA antibodies in the mucosal surface, as indicated by the existence of secretory IgA in stool samples	[17,18],
RotaRix	Rotavirus	Oral	Live attenuated monovalent vaccine: human VP7 and VP4 antigens from the G1P1 [8] strain	VP4 and VP7 specific humoral immune responses, mainly by the generation of mucosal IgA antibodies; strong protection against G1 and non-G1 serotypes (except in the G2 serotype), frequently associated with P [8]; poor VP7 specific cytotoxic T-cell immune response	[19,20]
RotaTeq	Rotavirus	Oral	Live attenuated pentavalent vaccine containing a mixture of five bovine–human reassortant rotaviruses: four express human VP7 (from strains G1, G2, G3, and G4) with VP4 from the bovine strain, and one expresses human VP4 (from the P1 [8] strain) with VP7 from the bovine strain; contains buffers to protect the viruses from gastric acid and a stabilizer solution (sucrose, sodium phosphate, sodium citrate, and polysorbate 80)	Generation of VP4 and VP7 specific antibodies, leading to robust heterotypic and homotypic neutralizing activity; high IgA seroconversion and systemic IgG antibodies; production of antibodies against nonstructural proteins, such as NSP2 and NSP4; strong protection against G1 and non-G1 serotypes (including the G2 serotype); enhanced levels of rotavirus-specific B cells expressing receptors for intestinal migration	[21,22]
Rotavac	Rotavirus	Oral	Live attenuated monovalent vaccine: human VP7 and VP4 antigens from the G9P [11] strain	Mucosal IgA and systemic IgG neutralizing antibodies specific for the multi-strain rotavirus	[23,24]
RotaSiil	Rotavirus	Oral	Live attenuated pentavalent vaccine containing a mixture of five bovine–human reassortant rotaviruses: VP7 gene from G1, G2, G3, G4, and G9 strains; thermostable vaccine		[25,26]
Biopolio (bOPV)	Poliovirus	Oral	Live attenuated bivalent vaccine containing a mixture of poliovirus 1 and 3 serotypes (Sabin strains); contains sucrose as a stabilizer	Generation of poliovirus IgG-specific antibodies in the serum and poliovirus IgA-specific antibodies in the mucosal linings of the intestines and respiratory tract	[27,28]
Vivotif	Typhoid fever	Oral	Live attenuated vaccine containing a *Salmonella typhi* Ty21a strain, in which enzymes essential for lipopolysaccharide biosynthesis were removed; despite this modification, the strain retains the capacity to produce sufficient amounts of LPSs, ensuring that antibodies are still produced against LPS antigens, thereby maintaining its protective properties	Strong humoral response illustrated by the generation of anti-*S. typhi* O-antigen (from LPSs) IgA and IgG antibodies in the gut and serum, respectively; robust cellular immune response distinguished by CD4^+^ and CD8^+^ T cells displaying gut-homing characteristics, resulting in the secretion of IFN-ɤ and cytotoxic T-cell activity	[29,30]
FluMist/Fluenz	Influenza type A and B viruses	Nasal spray	Live attenuated tetravalent vaccine with four hemagglutinin and neuraminidase antigens from circulating influenza virus strains: A strain—H1N1 and H3N2 and two B strains; contains an immunostimulant (arginine) and cryoprotectant (sucrose)	Cold-adapted vaccine designated to enable viral replication within the nasal passages while inhibiting replication in the lungs or other regions further down the body’s respiratory system; increased levels of secreted nasal IgA and systemic IgG antibodies; production of strong cell-mediated influenza specific IFN-ɤ^+^ T-cell responses and enhanced NK cell cytotoxicity activity	[31,32,33]
Nasovac-S	Influenza type A and B viruses	Nasal spray	Live attenuated trivalent vaccine: a strain—H1N1 and H3N2 and one B strain; contains several amino acids (L-Alanine, L-Histidine, and L-Arginine) as stabilizers	Robust immune response characterized by the synthesis of mucosal IgA antibodies targeting the two influenza surface proteins (hemagglutinin and neuraminidase), alongside systemic IgG antibodies in the bloodstream; potential CD4^+^ T-cell responses via IL-2 and IFN-ɤ production	[34,35,36]
Pandemic influenza vaccine H5Ν1-AstraZeneca	Influenza type A virus	Nasal spray	Live attenuated monovalent vaccine: a strain—H5N1; contains gelatin (porcine, type A), arginine hydrochloride, and monosodium glutamate monohydrate as stabilizers	Development of antibodies that effectively neutralized multiple clades of H5N1 viruses; possible activation of cellular immunity comparable to the response triggered by natural infection	[37]
iNCOVACC (BBV154)	COVID-19	Nasal drop vaccine	Adenovirus-vectored vaccine for SARS-CoV-2 that encodes a spike protein stabilized in its prefusion state, incorporating two proline modifications in the S2 subunit	Elevated neutralization titers against the original SARS-CoV-2 strain and cross-neutralizing responses to the Omicron BA.5 sub-lineage; high levels of specific IgA-secreting plasmablasts; strong T-cell-mediated immunity, despite a high baseline likely indicating previous adenovirus infection; presence of lung-resident lineage cells, particularly CD103^+^ CD69^+^ CD8^+^ T cells	[38]
Convidecia Air (Ad5-nCoV-IH)	COVID-19	Aerosolized vaccine for inhalation through the mouth	Recombinant adenovirus type 5-vectored vaccine that encodes the spike protein of SARS-CoV-2	Following a heterologous prime-boost regimen, there is a significant increase in neutralizing antibodies effective against various strains of SARS-CoV-2; promotes elevated sIgA levels and stimulates resident memory B and T cells in respiratory mucosa, enhancing local infection defense	[39,40]

**Table 2:** Overview of distinct macrophage phenotypes and their characteristic features and functions within the immune system and tissue microenvironment.

Macrophage Subtype	Stimulator Factors	Typical Receptors Expressed	Enzymes Expressed	Cytokines and Chemokines Secreted	Function
M1	LPS, IFN-ɤ, TNF-α, GM-CSF	MHCII, CD68, CD80, CD86, CD64	iNOS, IDO, ROS, MMPs	TNF-α, IL-1β, IL-6, IL-12, IL-23, CXCL5, CXCL9, CXCL10, CCL5	Pro-inflammatory activity Defense against infections and intracellular pathogens
M2a	IL-4, IL-13	CD163, CD206, CD209/DC-SIGN, Dectin-1, DCIR	Arginase-1, YM1	IL-10, TGF-β, CCL17, CCL22, CCL24	Anti-inflammatory activity Enhanced phagocytic capacity Tissue regeneration and repair
M2b	Immune complexes combined with TLR and/or IL-1 receptor agonists	CD163, CD206, CD209/DC-SIGN	Arginase-1	IL-10, TGF-β, IL-1, IL-6, TNF-α, CCL1	Anti-inflammatory activity Pro-inflammatory activity Modulation of immune responses
M2c	IL-10, TGF-β, glucocorticoid hormones	CD163, CD206, CD209/DC-SIGN, MerTK	Arginase-1	IL-10, TGF-β, CCL16, CCL18	Recruitment of immune cells Phagocytosis of apoptotic cells Supports angiogenesis
M2d	TLR or adenosine receptor agonists	CD163, CD206, CD209/DC-SIGN	Arginase-1	IL-10, TGF-β, VEGF, CXCL10, CXCL16, CCL5	Promotion of tumor progression Supports angiogenesis
Langerhans cells (LCs)	GM-CSF, TGF-β1, TLR agonists, TNF-α, IL-4	MHCII, Langerin/CD207, EpCam/CD326, CD1a, CD11c, CCR7, TLR2, TLR3, TLR5, TLR8, TLR9	IDO, Cathepsins, iNOS	IL-10, TGF-β, IL-23, IL-12, IL-6, TNF-α	Enhanced antigen presentation Immune surveillance Skin barrier maintenance

**Table 3:** Characteristic features of the different DC subsets, including conventional DCs (CD141^+^ DCs and CD1c^+^ DCs), plasmacytoid DCs (pDCs), and monocyte-derived DCs (Mo-DCs).

Dendritic Cell Subtype	Key Regulatory Molecules	Typical Receptors Expressed	Cytokines, Chemokines Secreted	Function
CD141^+^ DCs (conventional DCs)	IRF8, ID2, BATF3A	CD141, DNGR-1/CLEC9A, XCR1, Necl2/CADM1, CD80, CD11c, MHCII, TLR2, TLR3. TLR6, TLR8, TLR9	TNF-α, IL-1β, IL-6, IL-12, IFN-β, IFN-α, IL-15	Enhanced antigen presentation, particularly in the context of cross-presentation induction of CD8^+^ T-cell responses
CD1c^+^ DCs (conventional DCs)	IRF4, Notch2	CD206, CD11c, CD11b, CD1c, MGL/CD301, CD172a/SIRPα, MHCII, DCIR, Dectin-1, Dectin-2, TLR1, TLR2, TLR3, TLR4, TLR5, TLR6, TLR8	IL-10, TNF-α, IL-1β, IL-6, IL-12, IL-18, IL-15, IL-23	Enhanced antigen presentation to both CD4^+^ and CD8^+^ T cells Immunomodulation
Plasmacytoid DCs	IRFs	CD303, CD123, CD304, CD45RA, TLR7, TLR9	IL-10, IL-1β, IL-18, IL-6, TNF-α, IFN-λ, IFN-β, IFN-α	Producers of type I and III interferons Limited ability to present antigens Viral sensing
Monocyte-derived DCs	IRF4, IRF8, Notch2	CD64, CD11c, MHCII, CCR2, CD11b, CCR7, TLR2, TLR3, TLR4, TLR5, TLR7, TLR8, TLR9	IL-12, IL-6, IL-10, TNF-α, NO	Enhanced antigen presentation Immune regulation Initiation of adaptive immunity

## Data Availability

Not applicable.
